# CagA-dependent expression of anti-inflammatory cytokine IL-13 and TNFRSF member Fn14 in *Helicobacter pylori* infected gastric cells and tissues

**DOI:** 10.3389/fmicb.2026.1812604

**Published:** 2026-06-16

**Authors:** Rizki Amalia, Junko Akada, Ayush Khangai, Muhammad Asif Khan, Ricky Indra Alfaray, Kartika Afrida Fauzia, Tomohisa Uchida, Takashi Matsumoto, Ari Fahrial Syam, Muhammad Miftahussurur, Yoshio Yamaoka

**Affiliations:** 1 Department of Environmental and Preventive Medicine, Oita University Faculty of Medicine, Yufu, Japan; 2 Helicobacter pylori and Microbiota Study Group, Institute of Tropical Disease, Universitas Airlangga, Surabaya, Indonesia; 3 Research Center for Global and Local Infectious Diseases, Oita University, Yufu, Japan; 4 The Gastroenterology Center, The First Central Hospital of Mongolia, Ulaanbaatar, Mongolia; 5 Research Center for Preclinical and Clinical Medicine, National Research and Innovation Agency, Bogor, West Java, Indonesia; 6 Department of Advanced Medical Sciences, Faculty of Medicine, Oita University, Yufu, Japan; 7 Division of Gastroenterology, Department of Internal Medicine, Faculty of Medicine-Cipto Mangunkusumo Teaching Hospital, University of Indonesia, Jakarta, Indonesia; 8 Division of Gastroentero-Hepatology, Department of Internal Medicine, Faculty of Medicine, Dr. Soetomo Teaching Hospital, Universitas Airlangga, Surabaya, Indonesia; 9 Department of Medicine, Gastroenterology and Hepatology Section, Baylor College of Medicine, Houston, TX, United States

**Keywords:** CagA, chronic infection, cytokine network, Fn14, *Helicobacter pylori*, IL-13, inflammation

## Abstract

*Helicobacter pylori* infection is a major driver of gastric cancer, with the CagA oncoprotein promoting chronic inflammation and epithelial injury. However, the overall picture of the cytokine–receptor pathways involved in this process remains unclear. To investigate how CagA modulates epithelial signaling, we generated *cagA*-deletion and *cagA*-complemented mutants of the gerbil-adapted TN2 strain and infected AGS gastric epithelial cells for 6–48 h. We also analyzed 85 gastric biopsy specimens from a nationwide Indonesian survey. *In vitro*, wild-type and *cagA*-complemented *H. pylori* robustly induced IL-8, IL-1β, and TNF-α, as expected. In the CagA-positive strain, the newly identified anti-inflammatory cytokine, IL-13, was also induced. IL-13-related Fn14, a TNF receptor superfamily member, was strongly upregulated in a CagA-positive strain, whereas TNFR1 remained stable, and TNFR2 was consistently downregulated. In clinical specimens, Fn14 expression was elevated in atrophic gastritis and closely associated with *cagA* positivity. IL-13 expression showed a distinct pattern, increased in atrophic gastritis but unrelated to *H. pylori* or *cagA* status, instead correlating positively with TWEAK, the ligand for Fn14. Together, these findings suggest that cagA-positive *H. pylori* infection is associated with Fn14 upregulation, whereas *H. pylori* infection in general is linked to TNFR2 downregulation. IL-13-related signaling may instead reflect mucosal injury and repair responses, indicating context-dependent regulation of TNF receptor family pathways in gastric mucosal inflammation.

## 1 Introduction

Designated a Group 1 carcinogen by the World Health Organization, *Helicobacter pylori* (*H. pylori*) has become a major focus of research and clinical intervention due to its high global prevalence, established link to gastric cancer, and the unique opportunity for prevention through eradication therapy (Hooi et al., 2017). Infecting more than half of the world’s population, *H. pylori* initiates a pathological cascade—from chronic gastritis to atrophy, intestinal metaplasia, dysplasia, and carcinoma, which accounts for a substantial proportion of global gastric cancer cases (Hatakeyama, 2014). Among its virulence factors, CagA stands out as the sole effector protein delivered via the bacterium’s type IV secretion system (T4SS), conferring at least a tenfold increase in gastric cancer risk by reprogramming host cell signaling and inducing tumor-promoting inflammation (Ekström et al., 2001; Takahashi-Kanemitsu et al., 2019).

Once delivered into gastric epithelial cells via T4SS, CagA perturbs immune signaling, sensitizing cells to inflammatory stimuli by reducing IκB in a PAR1b-dependent manner and facilitating NF-κB activation through the c-Met–PI3K–AKT axis or TRAF6–TAK1 complex, leading to IKK activation and IκB degradation (Lamb et al., 2009; Suzuki et al., 2015; Takahashi-Kanemitsu et al., 2019). These events drive transcription of pro-inflammatory cytokines, including IL-1β, IL-6, and IL-8; IL-1β maturation is further promoted via NLRP3 inflammasome activation (Yong et al., 2015), while IL-6 triggers STAT3 signaling through gp130, sustaining inflammation and survival signals (Wang et al., 2023). Furthermore, CagA-positive *H. pylori* strains deliver other pathogen-associated molecular patterns, such as peptidoglycan and ADP-heptose, into epithelial cells via the secretion system, activating Nod1–NF-κB and ALPK1–TIFA pathways to further amplify cytokine release (Faass et al., 2021). Infiltrating immune cells in the gastric mucosa are also targeted, where CagA-dependent mechanisms stimulate NLRP3 inflammasome activity and IL-1β secretion (Takahashi-Kanemitsu et al., 2019).

This persistent, cytokine-driven inflammatory microenvironment fosters DNA damage, promotes epithelial proliferation, and synergizes with CagA’s direct oncogenic effects, ultimately accelerating progression from chronic gastritis to gastric cancer (Ma et al., 2016). Cytokines such as IL-8, IL-1β, and TNF-α are well-known for driving severe inflammation in *H. pylori* infection. However, additional cytokines with more subtle yet persistent roles in CagA-associated inflammation remain less explored. Beyond classical pro-inflammatory cytokines, emerging evidence suggests that epithelial injury and mucosal remodeling may involve cytokine–receptor networks that regulate both inflammation and tissue repair. IL-13 has been reported to mediate intestinal epithelial damage and barrier disruption, while the TWEAK/Fn14 pathway is associated with epithelial injury, inflammation, and tissue remodeling. Notably, IL-13-induced epithelial damage has been shown to require TWEAK and its receptor Fn14 in intestinal mucosal injury models, suggesting a potential functional connection between these pathways in gastric mucosal inflammation (Kawashima et al., 2011). Fn14, a TNF receptor superfamily member, can also activate NF-κB-related signaling and regulate cell survival or death responses through TWEAK/Fn14-dependent mechanisms (Wei-Lynn, 2008). However, whether IL-13 and Fn14 are involved in CagA-associated gastric epithelial responses remains unclear. In most studies investigating the inflammatory effects of CagA, attention has been focused on early, robust cytokine responses elicited under high bacterial loads, typically at multiplicities of infection (MOI) of 50–200. To identify cytokines that are not only strongly but also weakly and consistently regulated by CagA, we aimed to profile mRNA expression in host cells infected at lower *H. pylori* doses. This approach may reveal novel mediators involved in the sustained inflammatory state characteristic of chronic infection.

Because conventional *H. pylori* infection models using gastric epithelial cell cultures lack immune cell components, it remains difficult to assess the long-term, inflammation-related effects of CagA *in vitro.* To bridge this gap, we extended our analysis to human gastric biopsy samples, evaluating the expression of the screened cytokines in relation to *H. pylori* infection and *cagA* gene expression status. To explore these broad stages of infection, our study targets populations with low *H. pylori* prevalence, such as Indonesia, to identify new cytokine and receptor expression patterns that may distinguish inflammation predisposing to malignancy from benign inflammation. Integrating findings from cell-based infection models and infected patient tissue samples provided new insights into how CagA shapes host gastric immune responses during persistent *H. pylori* colonization. By identifying these molecular signatures, we aim to find new biomarker candidates of disease progression toward gastric cancer.

## 2 Materials and methods

### 2.1 Bacterial strain and culture

An East Asian-type *cagA*-positive *H. pylori* strain, TN2, derived from a Japanese patient (Watanabe et al., 1998), was re-adapted to Mongolian gerbils and stored in freezing medium at −80 °C. The bacteria were cultured on Brucella agar plates supplemented with 7% horse blood, 10 μg/mL vancomycin, and 2 μg/mL amphotericin B, incubated at 37 °C in a microaerophilic incubator.

### 2.2 Cell culture and *Helicobacter pylori* infection

A human gastric adenocarcinoma AGS cell was maintained and sub-cultured with RPMI 1640 (Nacalai Tesque, Kyoto, Japan) supplemented with 10% FBS (RPMI medium). The cells were cultured in a 12-well plate, with triplicate wells for each infection. *H. pylori* cultured on Brucella blood agar supplemented with 7% horse blood was diluted in RPMI 1640 medium, measured the OD590, and adjusted to MOI 10 (RNA analysis) and 50 (Western blotting analysis). The RPMI media of AGS cells were replaced with the media containing each MOI of *H. pylori* at the start of cell infection. After 6, 12, 24, 36, and 48 h of incubation, AGS cells were thoroughly washed once with PBS to remove residual media and debris.

### 2.3 Bacterial adaptation in Mongolian gerbils

The wild-type *H. pylori* strain TN2 and its derivative mutant strains (described below) were re-adapted to Mongolian gerbils after each genetic determination. *H. pylori* bacteria, previously grown for 3 days on Brucella blood agar, were transferred to 5 mL of Brain Heart Infusion (BHI) medium supplemented with 10% fetal bovine serum (FBS) in a 50 mL flask and pre-conditioned with 15% CO₂ gas. The cultures were incubated at 37 °C for 24 hours (H) with shaking and subsequently sub-cultured under the same conditions from a suspension adjusted to an OD₅₉₀ of 0.1. The OD_590_ 0.7–1.1 of a 1-day-cultured *H. pylori* (approximately 5–8 × 10^8^ CFU) suspension was inoculated by oral gavage and repeated once more after a 1-day interval. The gerbils were sacrificed at 2 weeks after the first inoculation. The stomach was carefully excised and rinsed with phosphate-buffered saline (PBS) to remove debris. The gastric fundus and residual contents were discarded, while the antrum and corpus were dissected and subsequently homogenized in PBS. The homogenized tissue was cultured on AccuRate Helicobacter plates (Shimazu, Kyoto, Japan) and incubated at 37 °C for 3–5 days under microaerophilic conditions. The *H. pylori* single colonies were isolated and cultured on Brucella blood agar, freeze-stocked after each specific check, and used for further examination (Supplementary Table S1) (Kubo et al., 2025).

### 2.4 Construction of *cagA* deletion strains, and the corresponding *cagA*-complemented strains

**Figure 1:**
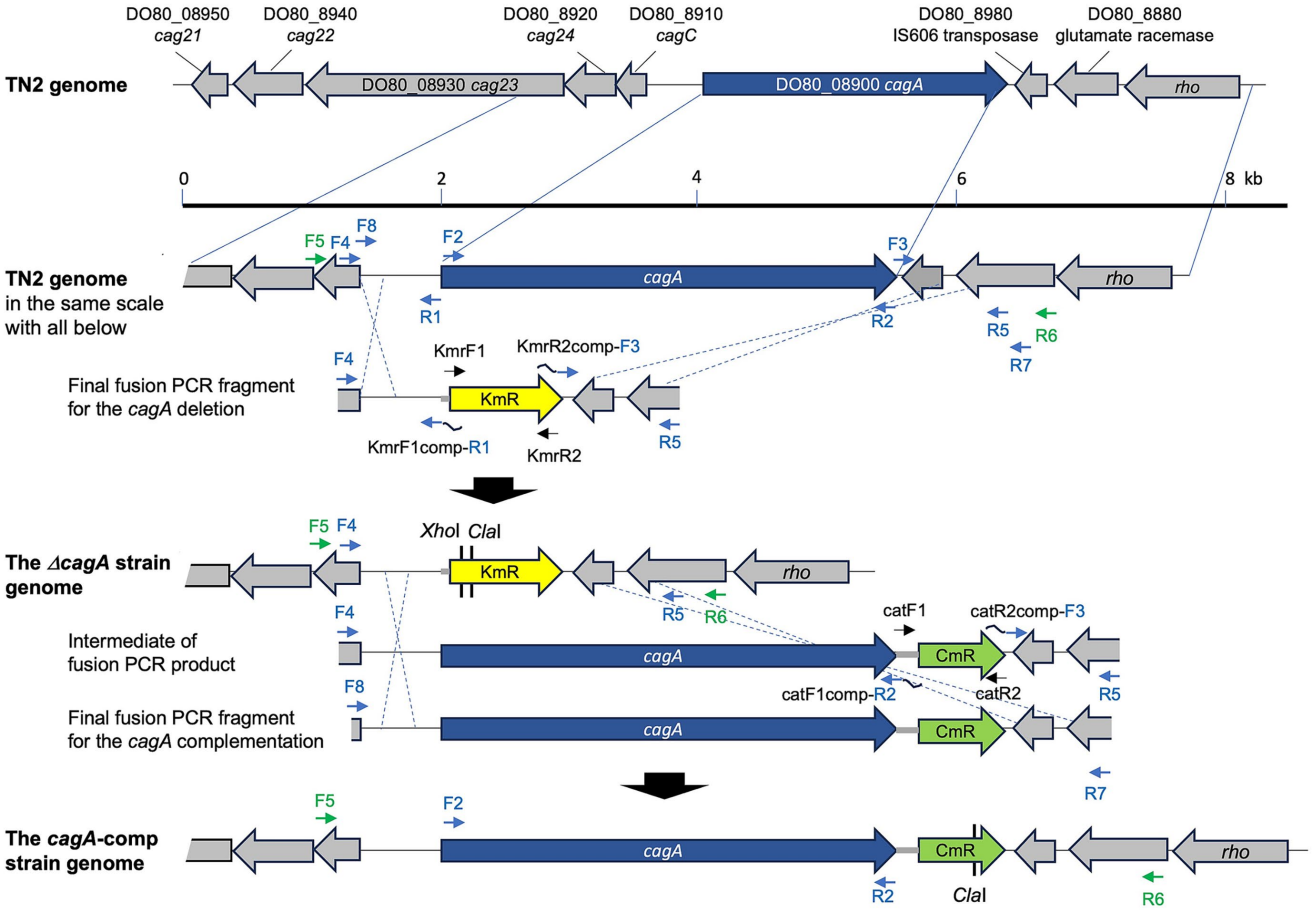
Schematic representation of the *cagA* region in strain TN2 genome and construction steps of *cagA* deletion (*ΔcagA*) and *cagA* complemented (comp) mutant strains. Outline of *cagA* deletion by replacing it with kanamycin resistance (KmR) cassette, and *cagA* gene complementation into the same genome location as TN2 with the downstream chloramphenicol resistance (CmR) cassette. The fusion PCR DNA fragments used for each transformation are shown with dotted lines showing the expected homologous recombination sites. Blue arrows: PCR primer used for construction, black arrows: primers for KmR or CmR cassettes, blue and black arrows: primers with flanking sequences, green arrows: outside checking primers.

The fusion PCR product for *cagA* deletion (*ΔcagA*) construct (F4/R5: 2.35 kb, Figure 1) consists of the fusion of three parts of PCR products using primers and DNA listed in Supplementary Table S2, following an allelic exchange strategy similar to that previously used for *H. pylori cagA* mutagenesis (Odenbreit et al., 2000). The kanamycin (Km) resistant (KmR) cassette (PCR product by primers KmrF1/KmrR2: 0.92 kb) containing promoter and ORF of aminoglycoside 3′-phosphotransferase gene (*aph*) from Tn903 were amplified from pUC-4 K plasmid, and were fused with upstream and downstream regions of *cagA* gene (F4/KmrF1comp-R1: 0.742 kb, and KmrR2comp-F3/R5: 0.735 kb, respectively) amplified from genomic DNA of *H. pylori* TN2 strain (GenBank accession No. AP09730). Both the upstream and downstream regions of the *cagA* gene were fused by PCR to the left and right sides of the KmR cassette, respectively. The entire fusion PCR process was performed using Primestar GXL DNA polymerase (TaKaRaBio, Shiga, Japan) according to the manufacturer’s instructions.

The resulting D*cagA* amplicon (F4/R5: 2.35 kb) was introduced into *H. pylori* TN2 via natural transformation, basically using methods previously described (Haas et al., 1993). Shortly thereafter, 100 ng of DNA was added to 1 mL of growing TN2 bacterial suspension culture at OD 0.1–0.2 in 6 H in Brucella broth supplemented with 10% FBS on a 6-well plate in a mixed-gas incubator. After overnight culture in the 6-well plate, portions of the culture were spread onto a Brucella agar plate supplemented with 10% FBS and 5 μg/mL kanamycin (Km) for 4 days. Colonies on a Km plate were individually picked onto a new plate, and the colony DNA was isolated by the phenol/chloroform method shortly thereafter. Successful full deletion of *cagA* and replacement of the kanamycin resistance cassette were confirmed by PCR using the outer primers (F4/R6: 2.39 kb; F5/R5: 2.50 kb). The amplified PCR product was also digested with the restriction enzyme XhoI at the specific site in the KmR cassette but not at any sites in the *cagA* gene or its upstream and downstream regions, and the expected fragment sizes were verified (Figure 1).

The fusion PCR product for *cagA* complementation (*cagA*-comp) construct (F8/R7: 5.72 kb) consists of the fusion of three parts of PCR products using primers and DNA listed in Supplementary Table S2. The chloramphenicol (Cm) resistant (CmR) cassette (cat-F1/cat-R2: 0.83 kb) containing promoter and ORF of chloramphenicol acetyltransferase (*cat*) gene from *Campylobacter jejuni,* were amplified from pBCS103 plasmid, and were fused with the upstream region and *cagA* gene ORF (F4/ catF1comp-R2: 4.26 kb), and its downstream region (catR2comp-F3/R5: 0.74 kb, respectively) amplified from genomic DNA of *H. pylori* TN2 strain, and fused by PCR at left side and right side of CmR cassette, respectively, one by one. The full-length fusion product (F4/R5: 5.78 kb) was cut and extracted after agarose gel electrophoresis, then amplified using nested primers to get a specific PCR product (F8/R7: 5.72 bp).

The resulting *cagA*-comp F8/R7 amplicon was introduced into the *ΔcagA* strain via the same natural transformation described above. However, using 250 ng of fusion DNA in a 6-well pre-culture of the *cagA* strain to complement the full-length *cagA* gene at the original genome location with a Cm cassette at the downstream. The transformants were selected on plates containing 5 g/mL chloramphenicol. Colonies were selected on chloramphenicol-containing plates and confirmed by no growth on kanamycin-containing plates. Integration of the complemented *cagA* gene and Cm resistance cassette was validated by PCR with outside primers (F5/R2: 5.06 kb, and F2/R6: 5.20 kb) and followed by restriction enzyme *Cla*I (Figure 1).

### 2.5 Confirmation of *cagA* gene and CagA protein sequence

The *cagA* region was further confirmed by Sanger sequencing for all *H. pylori* strains used in this study, including WT, *ΔcagA*, and *cagA*-complemented strains from both lab-grown and gerbil-adapted strains. Based on the TN2 reference genome sequence (LC007103.1), PCR amplification was performed using LongAmp Taq 2 × Master Mix (New England Biolabs, Ipswich, MA, USA) with the forward primer 5′-ATGGTGTAAATGGAACCCTAGTC-3′ and reverse primer 5′-TTAAGATTTCTGGAAACCACTTTTTGTAG-3′. The PCR products (1,023 bp) were examined by agarose gel electrophoresis with ethidium bromide staining to confirm the expected amplicon size before continuing to Sanger sequencing. The DNA samples were sent for Sanger sequencing to a commercial service (FASTMAC Co. Ltd., Kanagawa, Japan). The resulting FASTA sequences were aligned and translated to examine the CagA protein sequence, with particular focus on the EPIYA motifs.

### 2.6 Immunoblotting analysis

AGS cells at 70–80% confluence in 12-well plates were infected with *H. pylori* at an MOI of 50 for 24 h. The MOI of 50 was used specifically for immunoblotting to ensure sufficient detection of CagA and phosphorylated CagA signals, whereas lower MOI conditions were used for cytokine expression analyses to better evaluate CagA-associated responses under lower bacterial burden. Cells were thoroughly washed with PBS and lysed in 100 uL of lysis buffer (50 mM Tris–HCl, 150 mM NaCl, 1 mM EDTA, 1% Triton-X-100, 0.5% sodium deoxycholate), supplemented with Complete Mini (protease inhibitor cocktail, Merck, Darmstadt, Germany) and phosSTOP (phosphatase inhibitor cocktail, Merck). The lysates were subjected to Sodium dodecyl sulfate–polyacrylamide gel electrophoresis (SDS-PAGE) using Mini-protean TGX Gel (Bio-Rad, Hercules, CA, USA), and the gels were subsequently transferred onto polyvinylidene difluoride (PVDF) membranes (Merck). These membranes were used for immunoblotting analysis with the primary antibodies anti-phospho-tyrosine PY99 (Santa Cruz Biotechnology, Dallas, TX, USA), anti-CagA (Austral Biologicals, San Ramon, CA, USA), anti-UreB (Institute of Immunology, Tokyo, Japan), anti-actin (Santa Cruz), then peroxidase-conjugated 2ndary goat anti-mouse or anti-rabbit IgG antibodies (Jackson ImmunoResearch Laboratories, West Grove, PA). Detections were visualized using ImmunoStar-Zeta (Wako, Tokyo, Japan) under a chemiluminescence imager (ChemiDoc MP, Bio-Rad).

### 2.7 Clinical study population

A total of 85 RNA samples from gastric biopsy samples were randomly selected from 1,172 biopsies reported in a previous prospective nationwide survey across Indonesia and were designated for analysis of cytokine mRNAs. Only samples with sufficient RNA quality and availability of corresponding clinical and histopathological data were included in the present study. No additional selection based on *H. pylori* infection status, cagA status, or disease severity was applied. Biopsy collection and handling were described in our previous study (Fahrial Syam et al., 2015; Miftahussurur et al., 2015a, 2015b, 2017, 2021, 2023; Fauzia et al., 2020). Briefly, antral biopsy specimens were previously used for *H. pylori* culture, rapid urease test, histology, and mRNA analyses. Gastric biopsy tissues from patients were immersed in RNAlater (Ambion, Carlsbad, CA, USA) and stored at −80 °C.

### 2.8 Diagnosis by histology

Histological examination was performed based on the updated Sydney system for histological findings in the antrum, as described previously (Fahrial Syam et al., 2015; Miftahussurur et al., 2017, 2023). For further analysis, histological diagnoses were made by a single pathologist. The disease was characterized as follows: (1) normal, if none either or both of polymorphonuclear leucocyte (PMN) cells or mononuclear (MN) cells was found, with clearly separated glands from the submucosa by the muscularis mucosae; (2) gastritis, if either or both of PMN cells or MN cells was at least 1 grade, without Intestinal metaplasia (IM), or atrophy; (3) atrophic gastritis, if decreased or absent of normal gastric antral glands was found without IM, the grade was at least 1, regardless of the PMN and MN grades; and (4) IM, characterized by the alteration of gastric epithelial cells with intestinal-type epithelium, the was at least grade 1 regardless of the PMN, MN, or atrophy grades. In cases where grades were calculated for more than one of the aforementioned factors, the histological diagnosis was determined and prioritized according to disease severity: normal, gastritis, atrophic gastritis, and IM.

### 2.9 RNA extraction and reverse transcription-qPCR

Cultured Cells were washed with D-PBS and harvested with lysis Buffer RLT (QIAGEN, Venlo, Netherlands) with 10% 2-mercaptoethanol and stored at −80 °C, and extracted using RNA Mini Kit (QIAGEN). RNA from Gastric biopsy tissues of patients was extracted using the AllPrep DNA/RNA/Protein Mini Kit (QIAGEN) as in a previous study (Miftahussurur et al., 2023). Reverse-transcription (RT) PCR was performed with the SuperScript IV VILO Master Mix using 200 ng of total RNA concentration for cell culture and 4 ng/μL of total RNA concentration for biopsy sample as a template (ThermoFisher, Scientific, USA). Real-time PCR was performed using TaqMan® Gene Expression Assays (ThermoFisher). Triplet array plate, HUMAN CYTOKINE NETWORK was used under QuantStudio 6 pro real-time PCR system (ThermoFisher). The relative quantification of *IL-8, TNFa, IL-1β, IL-13*, *TNFRSF12A (Fn14)*, *TNFRSF1a (TNFR1)* and *TNFRSF1b (TNFR2)* mRNA levels was performed in duplex conditions. Specific probes were selected, the same probes used in the HUMAN CYTOKINE NETWORK array and HUMAN TNF SUPERFAMILY PATHWAY array of the TaqMan® Gene Expression Assays, and FAM/MGB-NFQ was selected as a reporter/quencher for IL-8, TNFa, IL-1, IL-13, Fn14, TNFR1, and *TNFR2*, whereas VIC-MGB-NFQ was selected for *GAPDH*. Reaction mixtures for PCR were prepared with mixing of synthesized cDNA of cell culture and biopsy samples as follows: TaqMan™ Fast Advanced Mix (Thermo Fisher Scientific), placed in a 96-well plate analyzer, and PCR was conducted according to the manufacturer’s instructions using CFX96 Real-Time PCR Detection System (Bio-Rad, Hercules, CA, USA). To normalize the targeted mRNA expression levels, the control *GAPDH* mRNA level was quantified simultaneously; the targeted mRNA level was presented as a ratio relative to GAPDH mRNA and calculated using the ΔΔCq method to obtain the relative value.

### 2.10 The RT-qPCR detection of *Helicobacter pylori* from the same biopsy samples

From the same extracted RNA obtained from gastric biopsy specimens, *H. pylori* infection was determined using HOT FIREPol® EvaGreen® qPCR Mix Plus (ROX) (Solis BioDyne, Tartu, Estonia). Extracted RNA was reverse transcribed to complementary DNA (cDNA). Quantitative PCR (qPCR) was performed using the CFX96 System (Bio-Rad). Melt curve analysis was conducted to confirm amplification specificity. Primer sequences targeting the *ureB* gene (three primer sets) and the *cagA* gene (two primer sets) are listed in Supplementary Table S3. Primers were designed based on the *ureB* and *cagA* sequences of *H. pylori* strain TN2 and also their consensus sequences of Indonesian clinical isolates (*n* = 34). The positivity in mRNA level was compared with conventional *H. pylori* detection methods (RUT, Giemsa-stained histology, culture, and serum IgG). Sample criteria of *H. pylori*-positive were considered by comparing with no amplification at 40 cycles from no RNA controls using each of the sets, if all three *ureB* primer sets amplified before 39 cycles, or at least two *ureB* sets before 38.5 cycles, or one *ureB* set before 36.5 cycles in a sample. The *cagA* positivity was defined as amplification of at least one *cagA* primer set before 38.5 cycles in a sample already positive for *H. pylori* by *ureB* described above.

### 2.11 Statistical analysis

A two-way analysis of variance (ANOVA) was conducted to evaluate the effects of incubation time and bacterial strain, as well as their interaction, on cytokine expression in the cell culture experiment. For clinical samples, the Kruskal–Wallis test was used to assess differences between diagnostic groups, followed by pairwise Mann–Whitney U tests. Statistical analyses and graphs were generated using R.

## 3 Results

### 3.1 Screening of *cagA-dependent* cytokine expression from AGS cells infected with MOI 10 of gerbils-adapted WT, *cagA*-full deletion strains in serum-containing media

**Figure 2:**
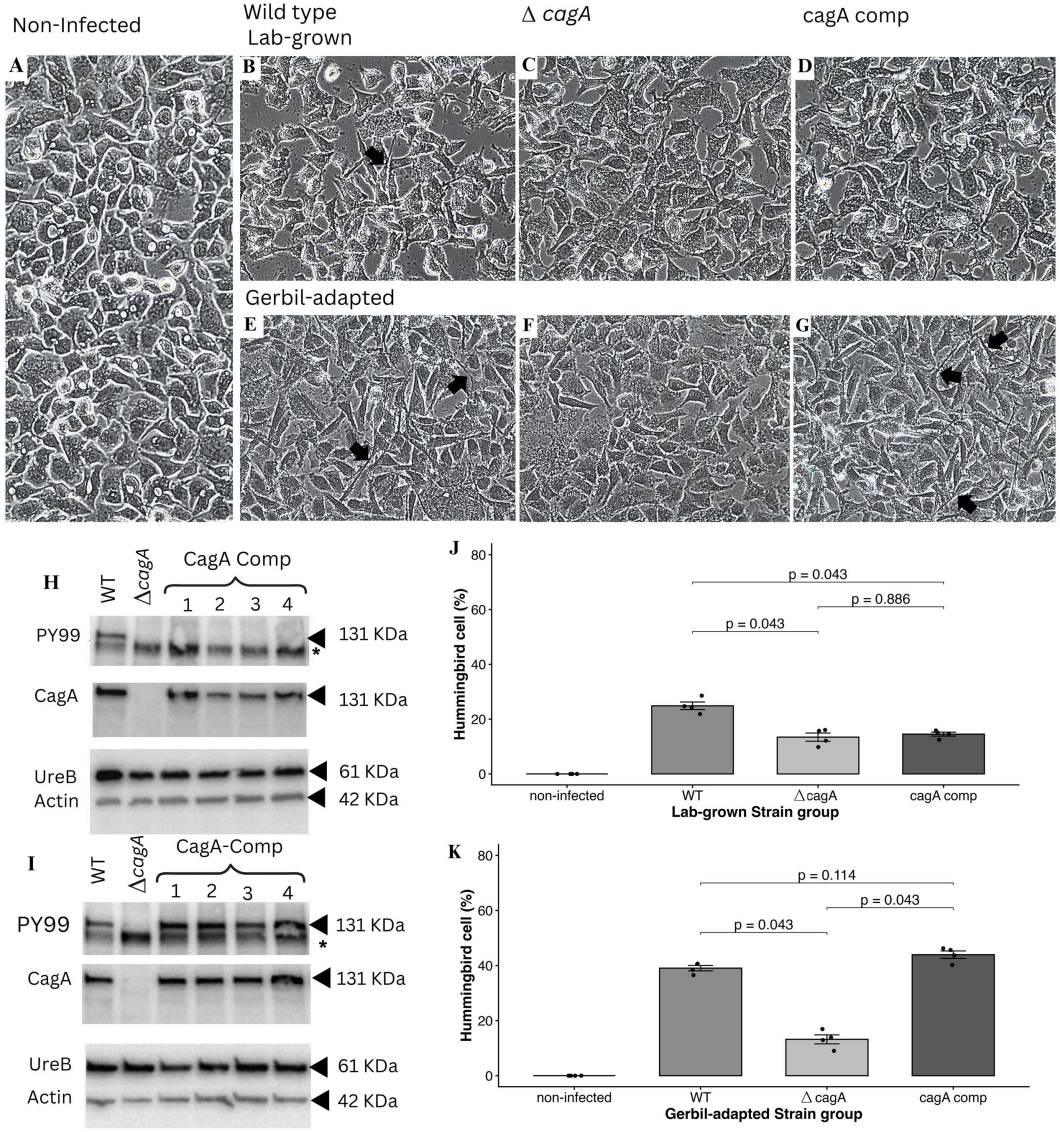
CagA phosphorylation–dependent induction of the hummingbird phenotype in AGS cells infected with lab-grown and gerbil-adapted TN2 strains. AGS cells were infected at a multiplicity of infection (MOI) of 50 for 24 h. **(A)** Non-infected AGS cells exhibited normal epithelial morphology. **(B)** Infection with the lab-grown wild-type (WT) strain induced the characteristic elongated “hummingbird” phenotype (black arrow). Conversely, infection with the lab-grown ΔcagA strain **(C)** and lab-grown *cagA*-complemented (*cagA*-comp) strain **(D)** did not induce the hummingbird phenotype, and cell morphology remained comparable to non-infected controls. **(E)** Infection with the gerbil-adapted WT strain induced pronounced cell elongation (black arrows). Cells infected with the gerbil-adapted ΔcagA strain **(F)** showed no hummingbird phenotype. Importantly, infection with the gerbil-adapted *cagA*-comp strain **(G)** restored the hummingbird phenotype (black arrows), similar to WT infection. **(H,I)** Immunoblot analysis of CagA phosphorylation. Phosphorylated CagA was detected using an anti-phosphotyrosine antibody (pY99) at approximately 131 kDa, corresponding to the CagA band detected by anti-CagA antibody. Phosphorylated CagA was not detected in cells infected with the lab-grown *cagA*-comp strain **(H)**, whereas clear phosphorylation signals were observed in cells infected with WT and all four gerbil-adapted *cagA*-comp strains **(I)**. UreB (61 kDa) and actin (42 kDa) were used as loading controls for bacterial and host cell proteins, respectively. The asterisk (*) indicates a non-CagA phosphorylated protein band detected in the pY99 blot. **(J,K)** Quantification of the hummingbird phenotype. In lab-grown strains **(J)**, the percentage of hummingbird cells in the *cagA*-comp group was comparable to that in the ΔcagA group (*p* > 0.05), indicating failure of phenotypic restoration. Conversely, in gerbil-adapted strains **(K)**, both WT and *cagA*-comp groups showed significantly higher proportions of hummingbird cells compared with the ΔcagA group (*p* < 0.05), with no significant difference between WT and *cagA*-comp.

To elucidate the functional role of CagA, we generated a fully cagA-deficient (*ΔcagA*) mutant and a *cagA*-complemented strain, in which *cagA* was restored at its native genomic locus, using both lab-grown and gerbil-adapted wild-type (WT) *H. pylori* TN2 strains (Figure 1). In AGS infection assays, the lab-grown WT strain induced the characteristic hummingbird phenotype, whereas the *ΔcagA* mutant failed to induce cell elongation. Notably, the *cagA*-complemented strain generated in the lab-grown strain did not restore the hummingbird phenotype, indicating impaired functional complementation of CagA (Figures 2A–D). Consistently, phosphorylation of translocated CagA was not clearly detected in cells infected with the lab-grown *cagA*-complemented strain (Figure 2H), and quantitative analysis confirmed that the proportion of hummingbird cells in this group remained comparable to that in the *ΔcagA* mutant (Figure 2J). Conversely, infection with the gerbil-adapted WT strain robustly induced cell elongation, while the gerbil-adapted *ΔcagA* mutant showed no hummingbird phenotype. Importantly, functional complementation of CagA was successfully restored in the gerbil-adapted *cagA*-complemented strain, as evidenced by reappearance of the hummingbird phenotype (Figures 2E–G), clear detection of CagA phosphorylation by immunoblotting (Figure 2I), and significantly increased proportions of hummingbird cells compared with the *ΔcagA* mutant (Figure 2K). To understand the reason why CagA phosphorylation was not restored in *the* lab-grown *cagA*-complemented strain but was restored in the gerbils-adapted *cagA-*complemented strain, the specific three EPIYA phosphorylation motifs-containing region of the *cagA* gene (1.023 kb) was amplified from all the strains used. As expected in the Δ*cagA* mutants from both lab-grown and gerbil-adapted strains, no PCR amplifications re-indicated the absence of the *cagA* amplicon (Supplementary Figure S1a). The EPIYA-regions of the WT and *cagA*-complemented strains from both lab-grown and gerbil-adapted strains were then sequenced, and all the *cagA* sequences were identical (Supplementary Figure S1b). However, the lab-grown *cagA*-complemented strain could not restore its CagA phosphorylation and related phenotypes. These findings suggest that restoration of CagA function may depend on additional bacterial or host-adaptation-related factors beyond the cagA sequence itself. Based on this result, the gerbil-adapted WT, *ΔcagA*, and *cagA*-complemented strains (Supplementary Table S1) were selected for subsequent AGS infection experiments.

**Figure 3:**
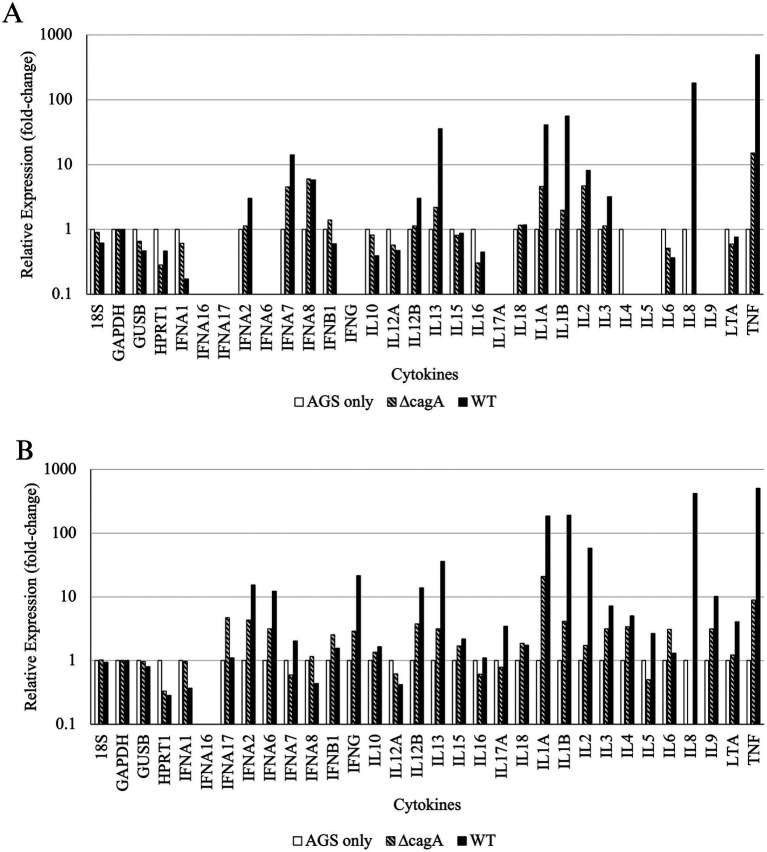
Cytokine array analysis of mRNA expression in AGS cells alone or infected with WT (TN2) or Δ*cagA H. pylori* (MOI 10) for 24 h. **(A)** Culture without FBS supplementation in RPMI1640 medium. **(B)** Culture using RPMI 1640 medium with 10% FBS supplementation. IL8 in each of the second panel (AGS infected with Δ*cagA H. pylori*) showed no reaction in both **(A)** and **(B)**, despite using the same cDNA sample in other cytokine wells. IL8 value of AGS infected with Δ*cagA H. pylori* was confirmed in Figure 4, comparing with WT, *cagA*-comp, and AGS only from a single experimental group.

To screen for modulation of unknown host cytokines related to CagA during infection, AGS cells were infected for 24H with either *H. pylori* WT or Δ*cagA* strain at an MOI of 10 with no-infected AGS as a control. The three sets of total RNA were analyzed for mRNA expression of cytokines using a Human Cytokine Network array (32 genes) in a 96-well panel. MOI of 10 was used to ensure a controlled, physiologically relevant *H. pylori* infection, minimizing host cell stress and cytotoxicity while maintaining cellular responses at 24H incubation. The presence of 10% fetal bovine serum (FBS) resulted in a cytokine expression profile broadly similar to that observed under serum-free conditions, but with a more robust response following *H. pylori* infection (Figure 3). To better approximate the physiological gastric microenvironment, all subsequent infection experiments were therefore performed under serum-supplemented conditions. In WT-infected AGS cells, canonical pro-inflammatory cytokines, including *IL-8*, *TNF*, and *IL-1β*, were markedly upregulated compared with non-infected controls and Δ*cagA*-infected cells. Conversely, Δ*cagA* infection induced only minimal changes in these cytokines, supporting the role of CagA in promoting inflammatory signaling. Notably, several less-characterized cytokines, including *IFNα2*, *IFNα6*, *IFN-γ*, *IL-12B*, *IL-13*, *IL-1α*, and *IL-2*, also demonstrated substantial induction (≥10-fold increase) in WT infection but not in Δ*cagA* infection (Figure 3B).

### 3.2 The *cagA*-dependent expression of anti-inflammatory cytokine IL-13 on AGS cell infection

Based on the cytokine array results, we were interested in the expression of IL-13, which appeared to be dependent on the presence of the *cagA* gene. Investigating IL-13 expression is also of interest because it has recently been reported as a target in cancer immunotherapy due to its role in promoting metaplastic epithelial changes associated with gastric cancer (Knudson et al., 2022; Noto et al., 2022).

**Figure 4:**
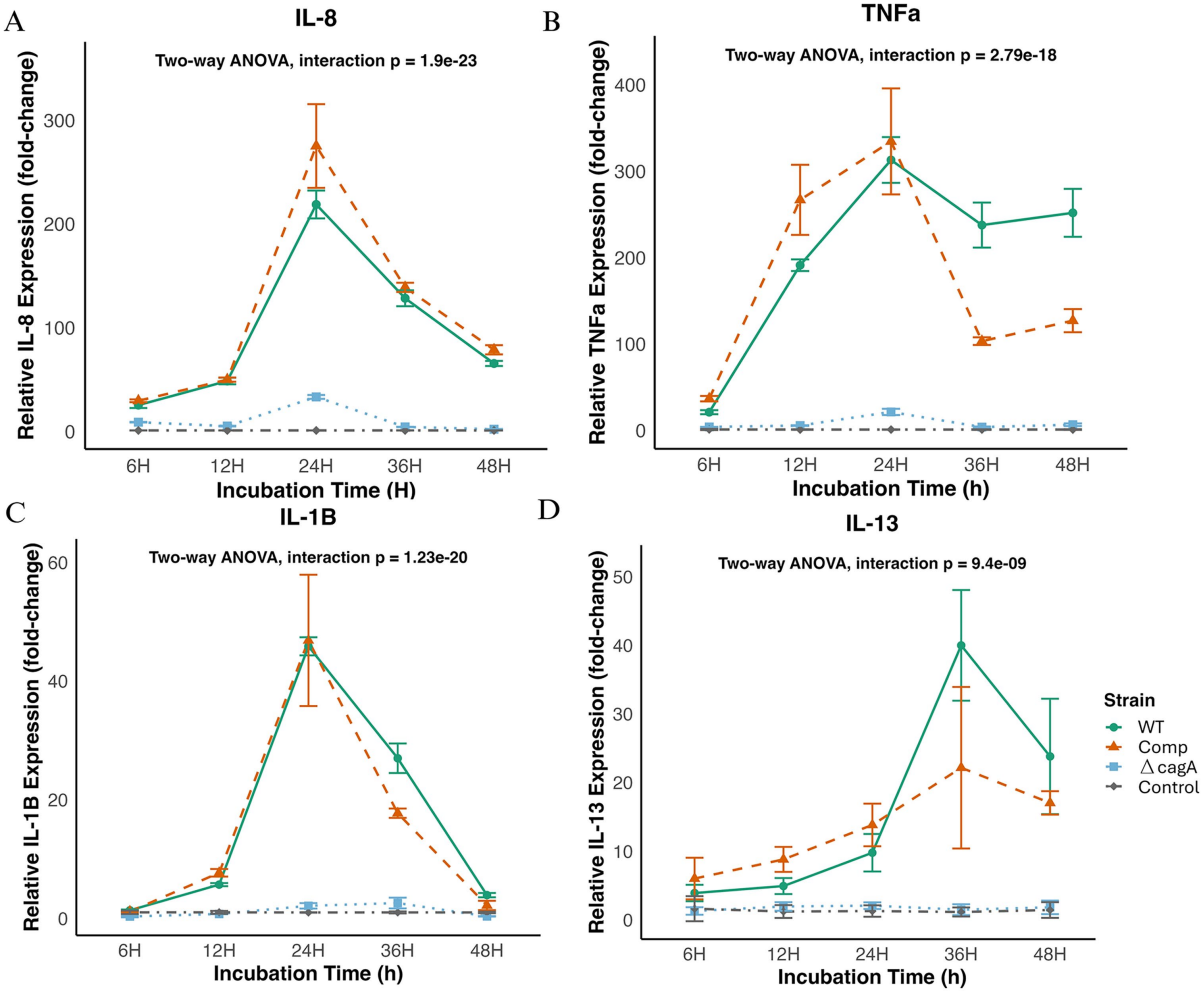
The expression time courses of selected cytokines in AGS cells infected (MOI 10) with *H. pylori* with or without *CagA*: **(A)** IL-8; **(B)** TNF-α; **(C)** IL-1β; **(D)** IL-13. The infection was performed at different incubation times (6 h, 12 h, 24 h, 36 h, 48 h) with *H. pylori* WT, *ΔcagA*, C*agA-*comp, and control (AGS only). Expression fold changes were adjusted with the control group to obtain the relative values. Data are presented as mean ± SD from three independent experiments. Statistical analysis was performed using Welch’s two-way ANOVA. Significant strain × time interaction effects were observed for all cytokines (*p* < 0.001).

The relative mRNA expression levels of IL-8, TNF-α, IL-1β, and IL-13 were confirmed and quantified at various incubation times post-infection with *H. pylori* at 10 MOI of WT, *ΔcagA*, and *cagA*-comp strains simultaneously (Figure 4). The time courses of IL-8, TNF-α, and IL-1β expression showed a peak at 24 h following infection with *cagA*-possessing WT and *cagA*-comp strains, but not with *cagA*, confirming their *cagA* dependency as expected, followed by a marked decline at 36H and 48 h. These temporal patterns are consistent with the roles of IL-8, TNF-α, and IL-1β as major pro-inflammatory cytokines activated during *H. pylori* infection (Figures 4A–C). Conversely, IL-13 expression (Figure 4D) exhibited delayed kinetics, with peak expression occurring at 36 H post-infection with *cagA*-possessing strains (x 20–40 folds in comp and WT infection), confirming their strict *cagA* dependency, followed by a decrease at 48H. This distinct temporal pattern suggests that IL-13 might be regulated by mechanisms distinct from those of the pro-inflammatory cytokines.

### 3.3 The gene expression of Fn14 and other TNFRs on AGS cell infection

The distinct time-course expression pattern of IL-13 observed from our AGS infection model in response to cagA-positive after *H. pylori* infection prompted us to further investigate the underlying mechanism. A previous study by Kawashima et al. (2011) suggested that IL-13 contributes to intestinal mucosal injury through the TWEAK–Fn14 pathway, raising the question of whether related components of this pathway might also be involved in the gastric epithelial context. Fn14 (Fibroblast growth factor-inducible 14), encoded by TNFRSF12A, belongs to the tumor necrosis factor receptor superfamily (TNFRSF) and serves as the receptor for TWEAK, a member of the TNF superfamily ligands. These membrane receptors bind TNF family ligands via extracellular cysteine-rich domains and play key roles in regulating inflammation, apoptosis, and tissue repair (Webster and Vucic, 2020; Li et al., 2025).

**Figure 5:**
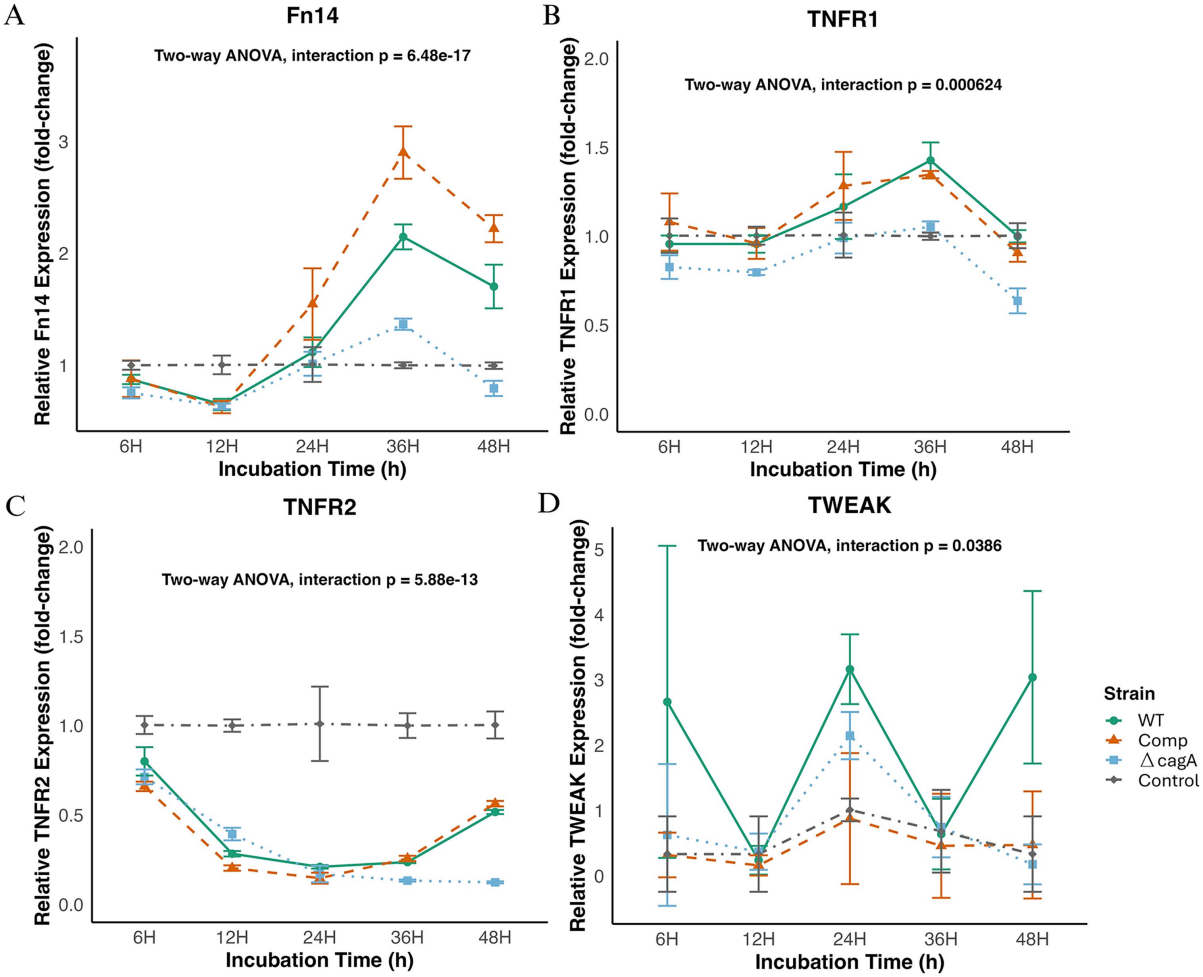
The expression time courses of TNF receptor superfamily in AGS cells infected (MOI 10) with *H. pylori* with or without *CagA*. **(A)** Relative mRNA expression levels of Fn14 (TNFRSF12A), **(B)** TNFRSF1A (TNFR1), **(C)** TNFRSF1B (TNFR2), and **(D)** Tumor Necrosis Factor-like Weak Inducer of Apoptosis (TWEAK) were measured at 6, 12, 24, 36, and 48 h post-infection in AGS cells infected with WT, *ΔcagA*, or C*agA*-comp *H. pylori* strains, or uninfected control. Expression values were normalized to control group levels and presented as fold changes. Data are presented as mean ± SD from three independent experiments. Statistical analysis was performed using Welch’s two-way ANOVA. Significant strain × time interaction effects were observed for all expressions (*p* < 0.001).

To examine this pathway in the context of *H. pylori* infection, we analyzed the mRNA expression of Fn14, along with two other TNFRSF members, TNFRSF1A (TNFR1) and TNFRSF1B (TNFR2), as control receptors, using the same RNA isolated from AGS cells infected with WT, *ΔcagA*, or *cagA*-comp *H. pylori* strains, as well as from non-infected controls (Figure 5). Infection with *H. pylori* strains induced a *cagA*-dependent upregulation of Fn14 (x 2–3 folds in WT and comp infections), particularly noticeable at 36H post-infection (Figure 5A). Conversely, TNFR1 expression was almost constant (x1.4-fold increase in WT infection at 36H as the highest), with slight dependence on *cagA* (Figure 5B); however, the TNFR2 expression was markedly downregulated following infection, and appeared independent of *cagA* (Figure 5C). Notably, expression of TWEAK, the ligand of Fn14, was difficult to monitor (Figure 5D). TWEAK expression showed substantial variability across time points, with some samples exhibiting very low or nearly undetectable fold-change expression. This may reflect the limited capacity of AGS epithelial cells to produce TWEAK under the present monoculture conditions. Therefore, TWEAK may be minimally expressed by gastric epithelial cells and may instead originate primarily from other cell types that are not represented in the AGS cell culture system.

Welch’s two-way ANOVA revealed statistically significant interaction effects for strain and incubation time on the expression of all three receptors (*p* < 0.001), indicating that both bacterial virulence and temporal dynamics contribute to their differential regulation.

### 3.4 Clinical samples and *Helicobacter pylori* status by RT-qPCR

A total of 85 Indonesian subjects were included in this study (Table 1). The demographic and histological characteristics of the cohort have been reported previously (Fahrial Syam et al., 2015; Miftahussurur et al., 2015a, 2017, 2021, 2023). The infection rate in the 85 samples was 42.4% by conventional methods. The study population included patients with gastritis, who were classified according to the Updated Sydney System, comprising normal mucosa (20/85; 23.5%), non-atrophic gastritis (7/85; 8.2%), atrophic gastritis (48/85; 56.5%), and intestinal metaplasia (10/85; 11.8%).

**Table 1:** Clinical demographic and conventional *H. pylori* status.

Demographic	Total samples	Conventional *H. pylori* status	
		Positive	Negative
N	85	36 (42.4%)	49 (57.6%)
*Age*			
Mean +SD	48.32 + 13.16	52.11 ± 11.56	46.09 ± 14.14
Min-Max	21–80	25–80	21–73
*Sex*			
Women	30 (35.3%)	14 (46.7%)	16 (53.3%)
Men	55 (64.7%)	22 (40.0%)	33 (60.0%)
*Diagnosis*			
Normal	20 (23.5%)	1 (5.0%)	19 (95.0%)
Non-atrophic gastritis	7 (8.2%)	2 (28.6%)	5 (71.4%)
Atrophic gastritis	48 (56.5%)	26 (54.2%)	22 (45.8%)
Intestinal metaplasia	10 (11.8%)	7 (70.0%)	3 (30.0%)

To connect simple *in vitro* cell infection experiments to a complex *in vivo* human biopsy cohort, the *H. pylori* infection status and *cagA* status for each biopsy sample need to be clearly defined. *Helicobacter pylori* infection status was usually determined using four conventional diagnostic methods, which were reported in a previous study (Fahrial Syam et al., 2015; Miftahussurur et al., 2015a, 2015b, 2021): serum anti-*H. pylori* IgG ELISA, rapid urease test (RUT), bacterial culture, and histopathological examination, each performed on separate gastric antral biopsy specimens, and a subject was classified as *H. pylori*-positive if at least one of these tests yielded a positive result. However, given the well-recognized patchy and heterogeneous distribution of *H. pylori* within the gastric mucosa (Pichon et al., 2020), bacterial colonization may vary even among biopsy specimens obtained from the same anatomical region. These phenomena may occur more often in biopsy samples from *H. pylori* low-prevalence areas, such as Indonesia, where a few *H. pylori* colonies are isolated from *H. pylori* positive samples but not from biopsy samples from patients with normal or mild gastritis, as in our experience. To minimize this potential sampling limitation and ensure the validity of our molecular analyses, RT-qPCR targeting a highly expressed gene in *H. pylori* should be effective with the same RNA extracted for cytokine expression profiling in this study. This approach was chosen to increase sensitivity and reduce sampling bias, as conventional diagnostic methods often rely on biopsy sites different from those used for RNA extraction.

**Figure 6:**
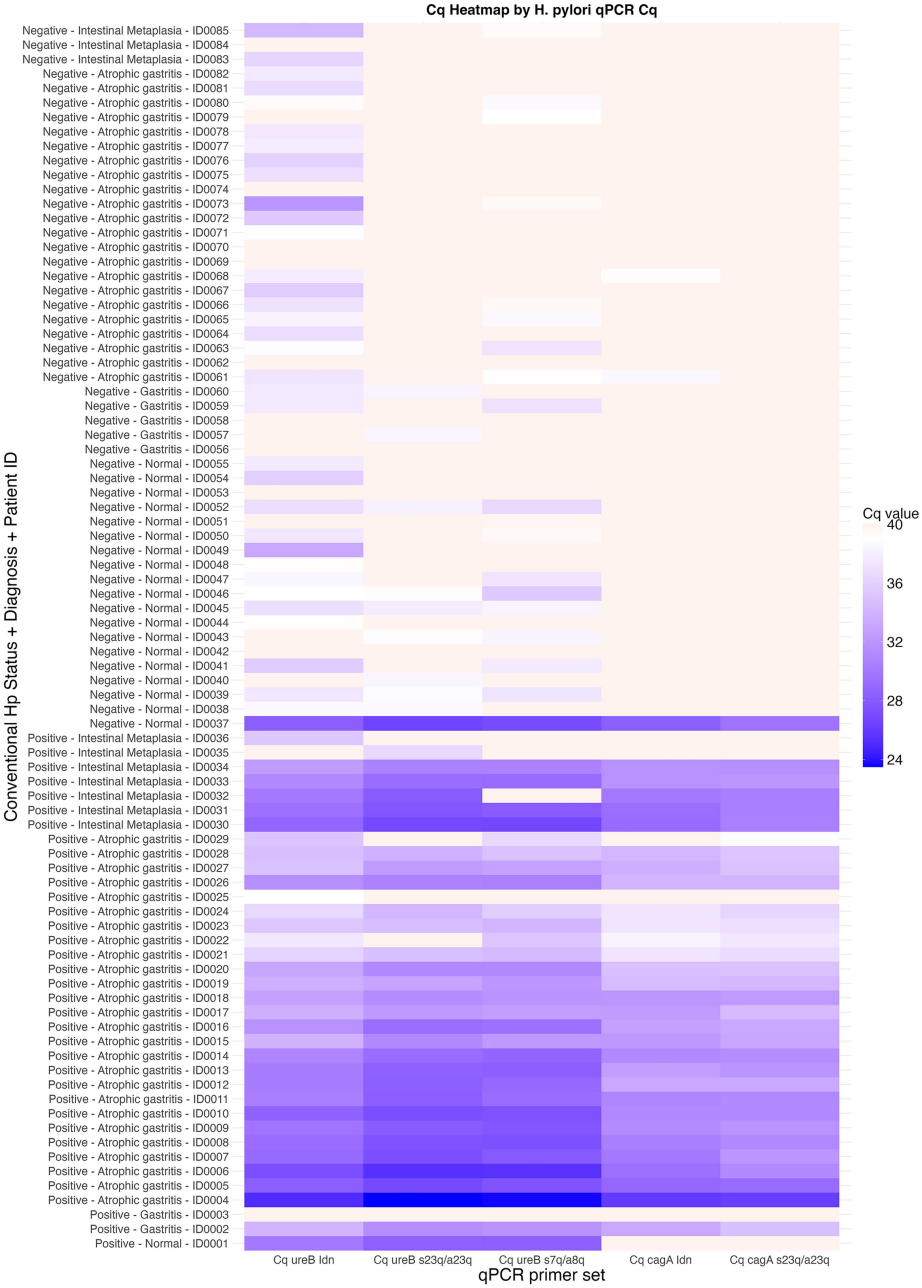
Heatmap of *H. pylori* Cq values by qPCR from all clinical biopsies. Light–deep violet colors indicate lower–higher Cq values, corresponding to higher amplification of the *ureB* and *cagA* genes. Conversely, cream–white colors represent higher Cq values, reflecting low to no amplification of these genes. Notably, some samples classified as *H. pylori*-negative by conventional methods still show deep violet coloration when tested with the Indonesian *ureB* primer set, indicating detectable *ureB* gene amplification in these conventionally negative patients.

We primarily determined RT-qPCR-based *H. pylori* positivity by targeting *ureB,* which encodes a subunit of urease, one of the most highly expressed proteins in *H. pylori*, and the *cagA* genes, using five newly designed primer sets (Supplementary Table S3) to overcome the *H. pylori* genome diversity. We used cDNA derived from the same gastric biopsy specimens that were later used for IL-13, FN14, and TWEAK expression analysis. Among the 85 samples, conventional *H. pylori* status was compared with *H. pylori* detection by RT-qPCR (Figure 6) as summarized in Table 2.

**Table 2:** Clinical characteristics of the study stratified by both conventional and qPCR *H. pylori* status.

*H. pylori* status	Total	Diagnosis			
		Normal	Gastritis	Atrophic gastritis	Intestinal metaplasia
Total	85	20 (23.5%)	7 (8.2%)	48 (56.5%)	10 (11.8%)
Conventional Hp infection status*					
Positive	36 (42.4%)	1 (5.0%)	2 (28.6%)	26 (54.2%)	7 (70.0%)
Negative	49 (57.6%)	19 (95.0%)	5 (71.4%)	22 (45.8%)	3 (30.0%)
*ureB* qPCR**					
*ureB* (+)	55 (64.7%)	11 (55.0%)	3 (42.9%)	32 (66.7%)	9 (90.0%)
*ureB* (−)	30 (35.3%)	9 (45.0%)	4 (57.1%)	16 (33.3%)	1 (10.0%)
*cagA* qPCR**					
*cagA* (+)	32 (37.6%)	1 (5.0%)	1 (14.3%)	25 (52.1%)	5 (50.0%)
*cagA* (−)	53 (62.4%)	19 (95.0%)	6 (85.7%)	23 (47.9%)	5 (50.0%)
Comparison of both positivity in qPCR and conventional method (%) with qPCR methods***					
Hp positivity	34/55 (61.8%)	1/11 (9.1%)	1/3 (33.3%)	25/32 (78.1%)	7/9 (77.8%)
*cagA* status	30/32 (91.7%)	0/1 (0%)	1/1 (100%)	24/25 (96.0%)	5/5 (100.0%)

*Conventional Hp infection status of each patient was determined using at least one of the four following methods: rapid urease test, Giemsa staining, and anti-*H. pylori* serum IgG antibody and *H. pylori* culture.

**ureB and cagA qPCR was done using the same cDNA used for cytokine qPCR analysis.

***The positivity of Hp and cagA status was evaluated from the positivity of the qPCR method and conventional, Hp positivity: conventional Hp positive & ureB qPCR (+)/ ureB qPCR (+); cagA status: cagA qPCR (+) & Hp conventional (+)/ cagA qPCR (+).

A total of 55 out of 85 subjects (64.7%) were *H. pylori* positive based on the detection of *ureB* expression by RT-qPCR, whereas 36 subjects (42.4%) were positive using conventional methods (Table 2), suggesting that this RT-qPCR offers improved sensitivity for identifying *H. pylori* infection. Interestingly, normal mucosa samples show that 55.0% (11/20) are *H. pylori*-positive by *ureB* expression, whereas conventional methods show only 5% (1/20) are *H. pylori*-positive, suggesting that RT-qPCR may improve *H. pylori* detection efficiency in samples with a lower number of *H. pylori* infections, such as normal samples. The highest proportion of qPCR-positive *H. pylori* was seen in intestinal metaplasia (90.0%, 9/10), followed by atrophic gastritis (66.7%), gastritis (42.9%), and normal mucosa (55.0%).

It is also important to evaluate the expression of the bacterial *cagA* gene using the same RNA of biopsy samples, because although 92% of *H. pylori* strains in Indonesia carry the *cagA* gene, serum anti-CagA positivity is only 78% and varies by geographic region (Miftahussurur et al., 2020).

The *cagA* expression-positive strains were most prevalent in the intestinal metaplasia (50%) and atrophic gastritis (52.1%) groups, compared with lower frequencies in gastritis (14.3%) and normal (5.0%) mucosa (Table 2). This pattern further supports the potential involvement of *cagA*-positive *H. pylori* in advanced gastric lesions. The overall *cagA* status with Hp conventional method positivity was almost more than 90%, equivalent to the *cagA* RT-qPCR method (Table 2), so together with the higher overall *ureB* expression detection by RT-qPCR, this supports RT-qPCR as a sensitive and reliable method for identifying *H. pylori* in the same biopsy tissue.

### 3.5 The IL-13 gene expression from clinical gastric samples

**Figure 7:**
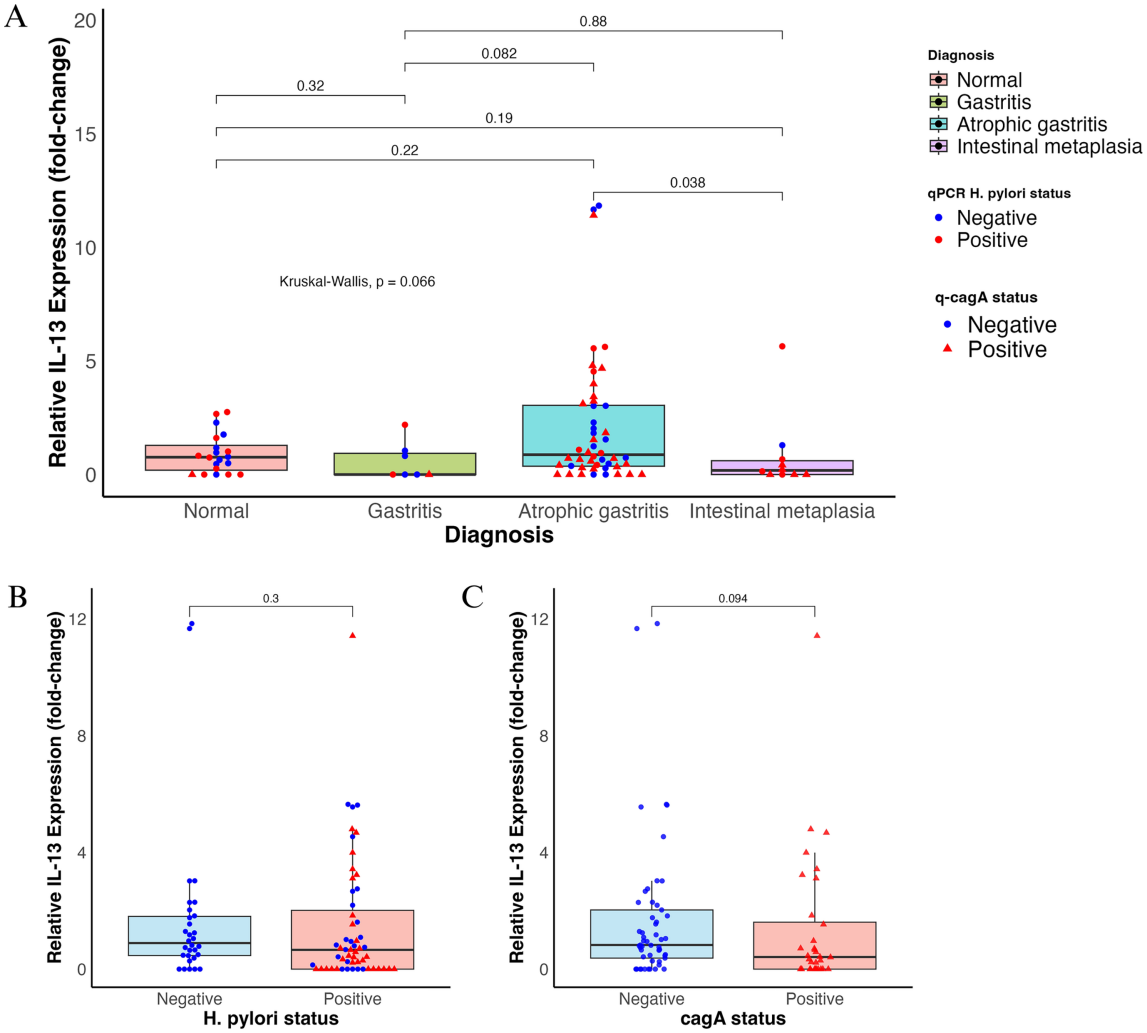
Relative IL-13 expression in gastric antrum tissue from clinical biopsy samples. **(A)** Boxplot showing IL-13 mRNA expression (fold-change) across histological diagnoses: normal, gastritis, atrophic gastritis, and intestinal metaplasia. A trend toward increased IL-13 expression was observed in atrophic gastritis, with a significant difference compared with intestinal metaplasia (*p* = 0.038; overall Kruskal–Wallis *p* = 0.066). *q*-*cagA* status is indicated by point shape (triangle: positive; circle: negative). **(B)** IL-13 expression according to qPCR-based *H. pylori* infection status by *ureB* expression (*q*-*H. pylori* status) and also indicated by qPCR-based *cagA* status (*q*-*cagA* status). No significant difference was found between *H. pylori*-positive and -negative samples (*p* = 0.72). **(C)** IL-13 expression stratified by *q*-*cagA* status. No significant difference was observed between *cagA*-positive and -negative groups.

IL-13 expression was investigated using the same set of 85 gastric antrum biopsies and analyzed as relative IL-13 mRNA levels (fold-change) stratified by histological diagnosis. Both the *H. pylori* infection status and *cagA* gene expression status were based on RT-qPCR detection (Figure 7). IL-13 expression tended to be higher in atrophic gastritis than in normal and other diagnostic categories (Figure 7A), although the overall difference did not reach statistical significance (Kruskal–Wallis, *p* = 0.066). A pairwise comparison showed that IL-13 expression was significantly higher in atrophic gastritis than in intestinal metaplasia (*p* = 0.038), suggesting a potential role for IL-13 during the transitional inflammatory stage prior to metaplastic changes. There were no significant differences in IL-13 expression between normal, gastritis, and atrophic gastritis (*p* > 0.05 for all comparisons). IL-13 expression was also compared between *H. pylori* status (Figure 7B). No statistical significance was observed (*p* = 0.72), although a tendency of increased IL-13 levels in *H. pylori*-positive samples was noted. Similarly, IL-13 expression did not differ significantly on *cagA*-status (*p* > 0.05) (Figure 7C). These results suggest that IL-13 may be moderately elevated or only transiently elevated in the antral mucosa during atrophic changes but not during the intestinal metaplasia stage.

### 3.6 The Fn14 and TWEAK gene expression from clinical gastric samples

**Figure 8:**
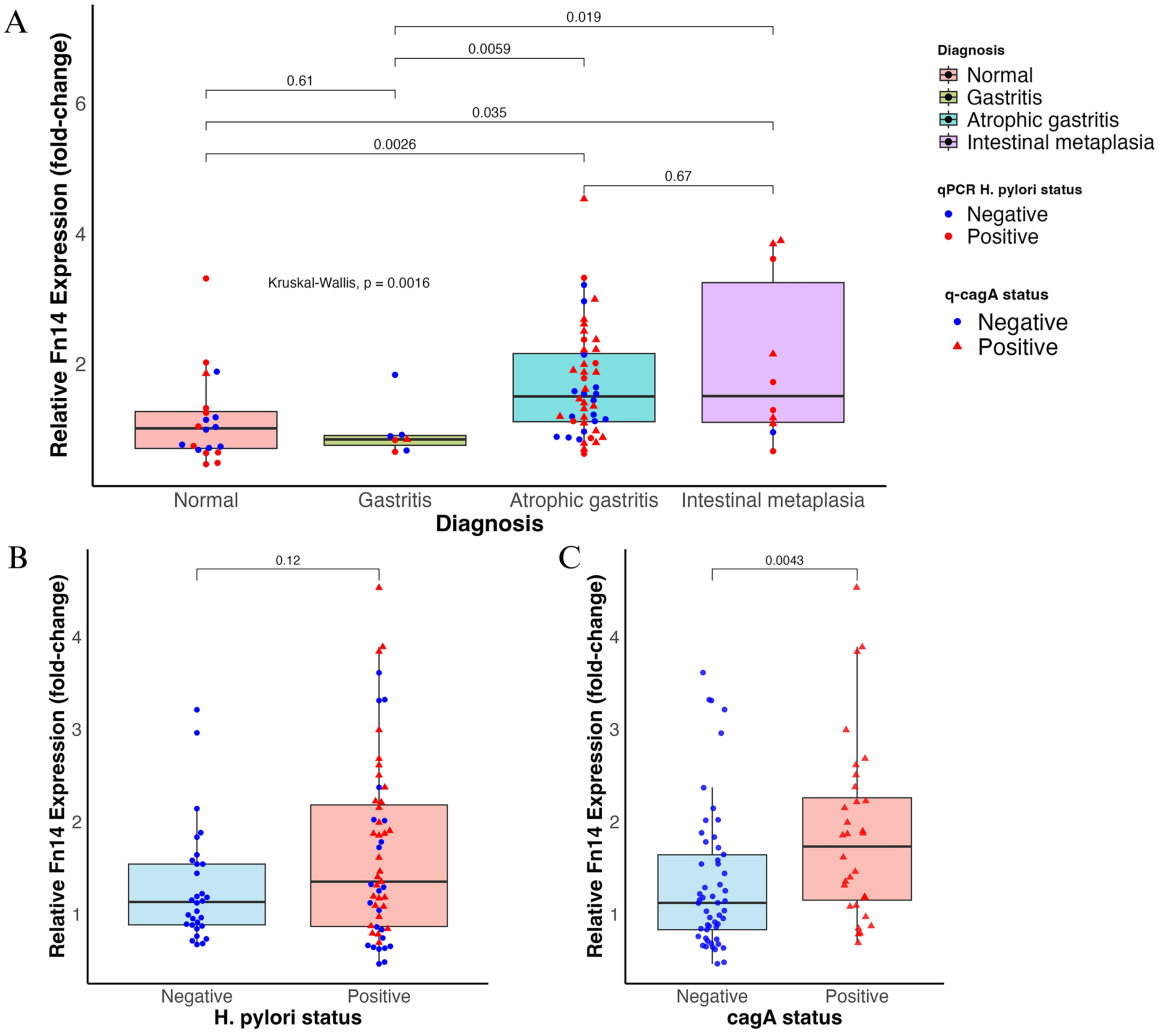
Relative expression of Fn14 from gastric antrum tissue of clinical biopsy samples. **(A)** Fn14 mRNA expression (fold-change) across different histological diagnoses: normal, gastritis, atrophic gastritis, and intestinal metaplasia. Fn14 expression was significantly elevated in atrophic gastritis and intestinal metaplasia compared to normal and gastritis (*p* < 0.05) (Kruskal–Wallis, *p* = 0.0016 overall). *q*-*cagA* status is indicated by point shape (triangle: positive; circle: negative). **(B)** Comparison of Fn14 expression according to qPCR-based *H. pylori* infection status by *ureB* expression (*q*-*H. pylori* status) and also indicated by qPCR-based *cagA* status (*q*-*cagA* status), showing a non-significant trend toward higher expression in infected individuals (*p* = 0.12). **(C)** Fn14 expression stratified by *q*-*cagA* status, revealing significantly higher expression in *cagA*-positive samples compared to *cagA*-negative samples (*p* = 0.0043).

**Figure 9:**
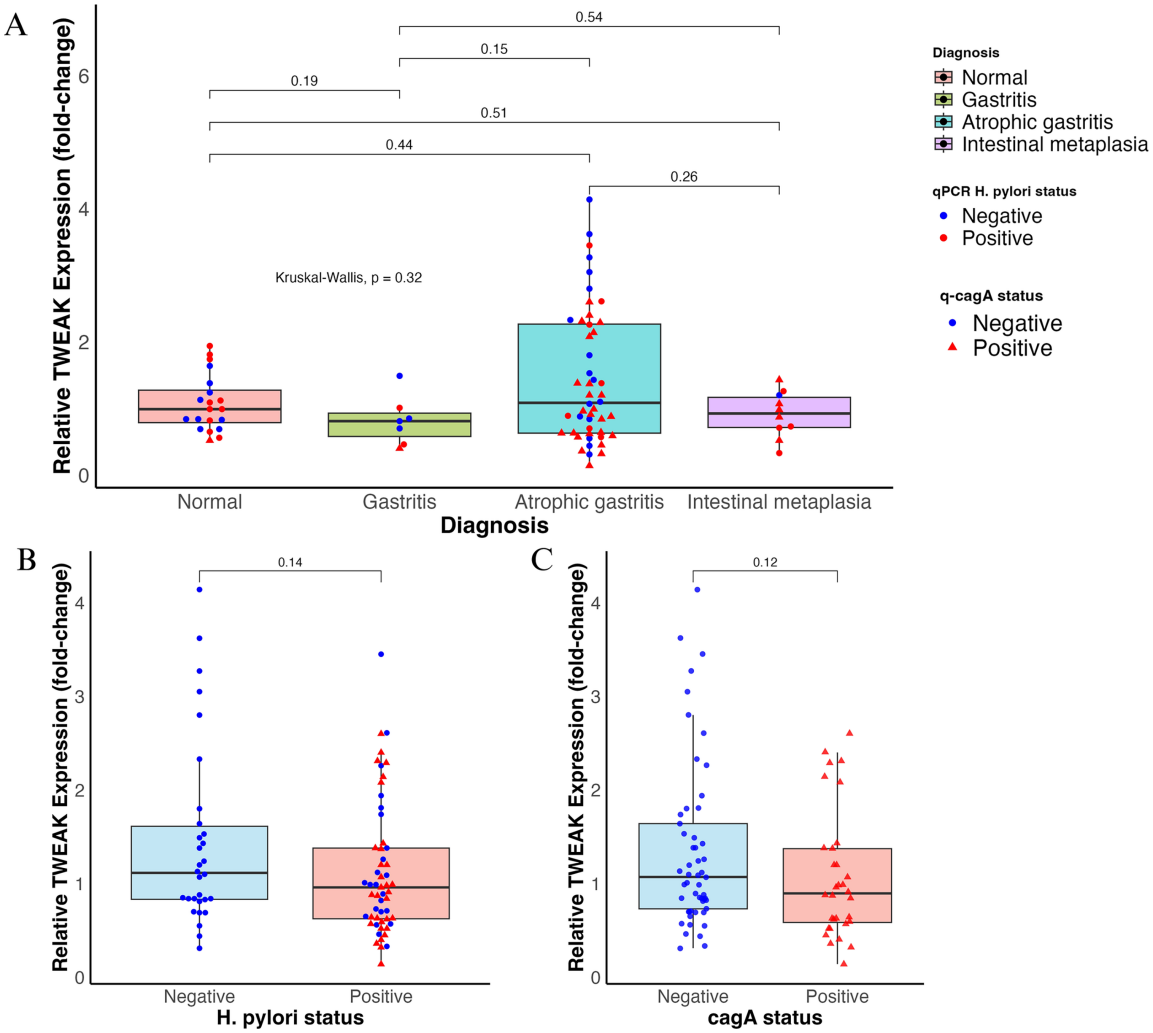
Relative expression of TWEAK from gastric antrum tissue of clinical biopsy samples. **(A)** mRNA expression (fold-change) of TWEAK across different histological diagnoses: normal, gastritis, atrophic gastritis, and intestinal metaplasia, showing no statistical difference across the diagnostic groups (*p* > 0.05) (Kruskal–Wallis, *p* = 0.32 overall). *q*-*cagA* status is indicated by point shape (triangle: positive; circle: negative). **(B)** TWEAK expression according to qPCR-based *H. pylori* infection status by *ureB* expression (*q*-*H. pylori* status) and also indicated by qPCR-based *cagA* status (*q*-*cagA* status). **(C)** TWEAK expression according to *q*-*cagA* status was not statistically different (*p* > 0.05).

The clinical relevance of Fn14 mRNA expression was also assessed in the same gastric antrum biopsies from patients, with a focus on its association with *H. pylori* status and *cagA* status (Figure 8). Fn14 expression significantly differed among diagnostic groups (Kruskal-Wallis, *p* = 0.0016) (Figure 8A). The highest expression was observed in patients with atrophic gastritis, which showed significantly increased Fn14 levels compared to normal (*p* = 0.0026) and gastritis (*p* = 0.0059), as well as in intestinal metaplasia, which showed significantly increased levels compared to normal (*p* = 0.035) and gastritis (*p* = 0.019). Regardless of overall *H. pylori* status, Fn14 expression (Figure 9B); however, importantly, when stratified by *cagA* status, Fn14 expression was significantly elevated in patients infected with *cagA*-positive strains compared to those not infected and infected with *cagA*-negative strains (*p* = 0.0043) (Figure 8C). This indicates that Fn14 induction is more strongly linked to *cagA*-mediated virulence mechanisms than to the presence of *H. pylori* infection alone. Taken together, these findings suggest that Fn14 contributes to the progression of atrophic changes through pathways related to *cagA*-dependent epithelial activation.

The potential involvement of Fn14 signaling in gastric mucosal alterations was examined by the mRNA expression of TWEAK, the ligand of Fn14 (Figure 9). TWEAK expression was consistently detectable in gastric tissue RNA samples and was modestly elevated in cases of atrophic gastritis compared with normal mucosa, gastritis, and intestinal metaplasia (Figure 9A); however, this difference did not reach statistical significance (Kruskal–Wallis, *p* = 0.32). Further stratification by *H. pylori* infection status (Figure 9B) and *cagA* positivity (Figure 9C) also revealed no significant differences in TWEAK expression (*p* = 0.14 and *p* = 0.12, respectively). Therefore, we observed atrophy-dependent, *cagA*-dependent Fn14 expression in gastric tissues but not the expression of its ligand, TWEAK.

### 3.7 The correlation between IL-13 and TWEAK gene expression

**Figure 10:**
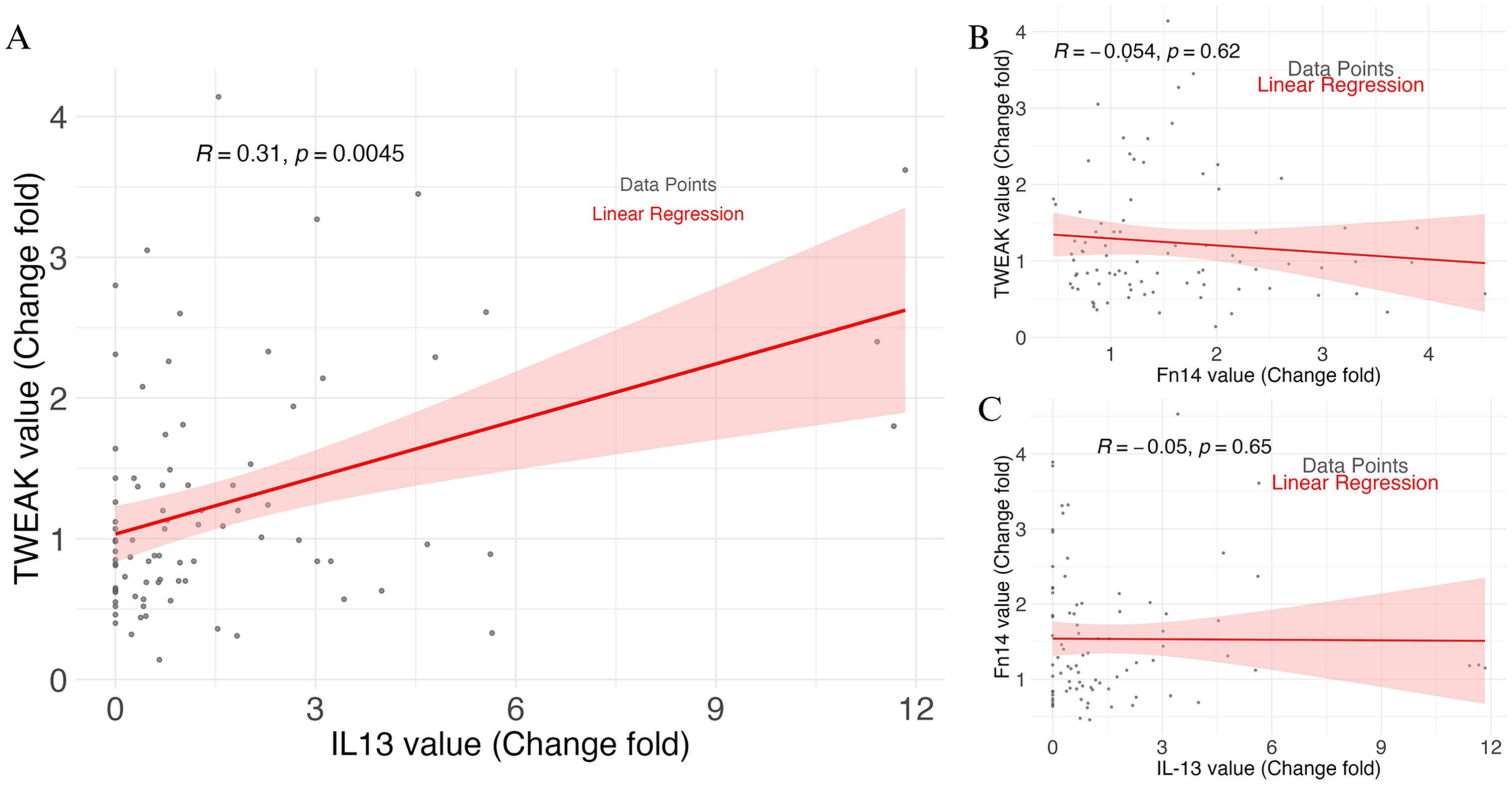
Correlation analysis between IL-13, TWEAK, and Fn14 mRNA expression in gastric antrum biopsies. **(A)** A statistically significant positive correlation was observed between IL-13 and TWEAK mRNA expression levels (*R* = 0.31, *p* = 0.0045), suggesting that IL-13 may contribute to the upregulation of TWEAK in the gastric mucosa. **(B)** No significant correlation was found between TWEAK and Fn14 expression (*R* = −0.054, *p* = 0.62). **(C)** Similarly, no correlation was observed between IL-13 and Fn14 expression (*R* = −0.05, *p* = 0.65). Each panel shows individual data points with a linear regression line (red) and 95% confidence interval (shaded area). Correlations were calculated using Spearman’s rank test.

The interplay between IL-13 and the TWEAK–Fn14 axis in gastric tissues was evaluated using pairwise Spearman correlation analyses of relative mRNA expression levels in gastric antrum biopsy samples to understand better the direction of their association (Figure 10). A statistically significant positive correlation was identified between IL-13 and TWEAK expression (*R* = 0.31, *p* = 0.0045; Figure 10A), indicating that IL-13 expression may contribute to the transcriptional upregulation of TWEAK in the gastric biopsy sample. By contrast, no significant correlation was observed between TWEAK and Fn14 expression (*R* = −0.054, *p* = 0.62; Figure 10B), nor between IL-13 and Fn14 (*R* = −0.05, *p* = 0.65; Figure 10C). The association between IL13 expression and TWEAK expression levels supports a possible mechanistic link between cytokine signaling and TWEAK expression in the gastric mucosa across different cell types.

## 4 Discussion

The *cagA* complemented mutant strain was generated to confirm that any phenotypic changes observed in the *ΔcagA* mutant were specifically attributable to the loss of *cagA*, rather than to polar effects on adjacent genes or defects in the type IV secretion system (T4SS) (Kubo et al., 2025). Notably, functional restoration of CagA activity was achieved only in the gerbil-adapted TN2 strain, whereas the lab-grown TN2 strain failed to fully recover CagA-dependent phenotypes following complementation. Previous genome-wide analyses have shown that gerbil adaptation of *H. pylori* is primarily associated with genetic diversification during *in vivo* infection without clearly defined global alterations in virulence phenotypes (Suzuki et al., 2019). Infection of AGS cells with the gerbil-adapted wild-type and cagA-complemented strains induced the characteristic hummingbird phenotype and Src/Abl-dependent phosphorylation of translocated CagA (Bourzac et al., 2007a; Tegtmeyer et al., 2017), whereas in the lab-grown TN2 strain, this phenotype was observed only with the wild-type strain. The identical *cagA* region sequences observed in all *cagA*-positive strains from both the lab-grown and gerbil-adapted strains (Supplementary Figure S1b) indicate that the lack of CagA phosphorylation in the complemented strain derived from the lab-grown culture was not attributable to failed *cagA* re-insertion or defects in the *cagA* sequence itself. These findings suggest that CagA-dependent phenotypes may be influenced by additional bacterial or host-adaptation-related factors beyond the presence of the cagA gene alone. Host-driven adaptation may affect processes such as protein translocation, phosphorylation efficiency, or downstream signaling responses, rather than directly altering the *cagA* sequence itself. This interpretation is supported by the identical *cagA* sequences observed among all cagA-positive strains, together with previous genome-wide analyses showing that gerbil adaptation is associated with mutations in genes involved in colonization, chemotaxis, pH regulation, and outer membrane functions (Suzuki et al., 2019). Accordingly, conclusions regarding CagA-dependent effects in this study are primarily based on observations in the gerbil-adapted strain, in which functional complementation was consistently achieved.

To characterize early signaling and transcriptional regulatory responses following infection, this study examined the expression of the anti-inflammatory cytokine IL-13 at the mRNA level. We found that IL-13 transcription is upregulated in AGS cells infected with *H. pylori* cagA-positive strains, a response that remains insufficiently defined in the context of early host–pathogen interactions. Alongside IL-13, we also observed the upregulation of well-known pro-inflammatory cytokines IL-8, TNF-α, and IL-1β, consistent with previous findings in *H. pylori*–infected gastric tissues (Miftahussurur et al., 2023; Yang et al., 2024), and *cagA-*dependent experimental cell infection using WT and its *cagA*-deletion or mutant *cagA* strains for IL-8 (Backert et al., 2001). IL-13 is best known as a type 2 cytokine produced by T helper 2 (Th2) cells that protects against parasitic infections and regulates allergic inflammation alongside IL-4 (Bernstein et al., 2023). In AGS cells infected with *H. pylori*, we found that gastric epithelial cells expressed IL-13 in a *cagA*-dependent manner. Although the AGS cell model provides a useful *in vitro* platform for evaluating CagA-dependent signaling and morphological changes, it does not fully reflect the complexity of the *in vivo* gastric niche, where host-driven adaptation, immune responses, and microenvironmental factors may substantially influence bacterial functionality and infection outcomes (Odenbreit et al., 2000; Bourzac et al., 2007b; Kinoshita-Daitoku et al., 2021). Furthermore, cytokine expression analyses were performed at a single lower infection dose (MOI 10), which was selected to minimize excessive host cell stress and to allow detection of subtle CagA-associated cytokine responses. Conversely, a higher MOI (50) was used only for immunoblotting and morphological assays to ensure sufficient detection of CagA phosphorylation and cell elongation phenotypes. Therefore, future dose–response analyses across multiple MOIs would help clarify whether these cytokine responses are dose-dependent.

The present study combined an *in vitro* epithelial infection model with clinical gastric biopsy analysis to distinguish epithelial cell–specific responses from tissue-level mucosal responses. The AGS cell model allowed us to examine the direct effect of CagA on gastric epithelial signaling under controlled conditions. In this system, *cagA*-positive strains strongly induced Fn14 expression and altered TNF receptor family responses, including the downregulation of TNFR2. These findings suggest that CagA can directly modulate epithelial cytokine–receptor signaling. However, the clinical biopsy specimens represent a more complex mucosal environment composed of epithelial cells, immune cells, stromal cells, and inflammatory infiltrates. Therefore, the biopsy data provide important translational context for determining whether the epithelial responses observed *in vitro* are reflected in human gastric tissue.

In clinical gastric mucosal tissues, IL-13 expression was elevated in atrophic gastritis and appeared lower in intestinal metaplasia compared to atrophic gastritis, although it was not significantly different from normal tissue. However, the origin of IL-13–expressing cells in clinical gastric samples is unclear in this study, Although our *in vitro* results suggest that gastric epithelial cells are capable of expressing IL-13, immune cells within the gastric microenvironment may also contribute to its expression, particularly because two of the three atrophic gastritis samples with the highest IL-13 expression (11–12-fold) were *H. pylori*-negative (Figure 7). Previous studies have shown that IL-13 produced by group 2 innate lymphoid cells (ILC2s) directly affects gastric epithelial cells and promotes the development of spasmolytic polypeptide-expressing metaplasia (SPEM), a precursor to gastric carcinoma (Contreras-Panta et al., 2024). In addition to ILC2s, mast cells, macrophages, T cells, and B cells, IL-13 is expressed in the mouse gastric mucosa, where it acts directly on epithelial cells to drive metaplastic development (Noto et al., 2022). The moderately elevated IL-13 levels (4–6-fold) observed in the *H. pylori*–positive atrophic gastritis tissue samples in this study (Figure 7) may support a role for IL-13 in the early metaplastic transition in some cases of atrophic gastritis. IL-13 signals through IL-4R*α*-containing receptor complexes, activating JAK kinases and inducing STAT6 phosphorylation, which drives transcriptional programs involved in immune regulation, epithelial differentiation, and inflammatory remodeling (Gordon and Martinez, 2010; Bao and Reinhardt, 2015). In parallel, epithelial stress and inflammatory cues may promote activation of the TWEAK–Fn14 axis, a signaling pathway known to regulate tissue repair, cytokine amplification, and NF-κB–dependent inflammatory responses (Burkly et al., 2011).

Previous studies have shown that IL-13-induced epithelial damage requires the presence of Fn14 (Kawashima et al., 2011). Therefore, it is plausible that tissues in which Fn14 is induced, such as the injured gastric mucosa, may become more susceptible to IL-13-mediated epithelial injury during *H. pylori* infection. In the present study, Fn14 expression showed a similar dependence on *cagA* expression in both the *in vitro* model and clinical gastric biopsy samples, consistent with previous reports linking Fn14 expression to epithelial injury responses (Dohi et al., 2009; Abu Bakar et al., 2023). IL-13-driven up-regulation of Fn14 can enhance TNF-α-mediated signaling through TNFR1 and TNFR2, thereby amplifying downstream inflammatory responses (Kawashima et al., 2011; Hu et al., 2017). Previous studies have suggested that IL-13 may interact with Fn14-related pathways; however, such regulatory relationships were not directly demonstrated in the present study. In our study, Fn14 expression showed a cagA-associated upregulation pattern in both *in vitro* and clinical samples, whereas TNFR2 expression is broadly downregulated following *H. pylori* infection, and TNFR1 expression remains relatively unchanged. TNFR2 is known for its cytoprotective effect by promoting cell survival through TRAF2/cIAP1/2 recruitment and NF-κB activation (Siegmund et al., 2023). Thus, the observed downregulation of TNFR2 across infection conditions suggests that *H. pylori* may weaken epithelial pro-survival signaling. Because TNFR2 primarily mediates cytoprotective NF-κB activation, its reduction may shift the balance toward TNFR1-driven cytotoxic pathways in the presence of abundant TNF-α. This interpretation is consistent with previous findings showing that loss of TNFR2 reduces the expression of survival genes and sensitizes gastric epithelial cells to apoptosis (Rossi et al., 2022). Conversely, Fn14—a non-death receptor that modulates cell fate by sequestering TRAF2/cIAP1/2 (Wei-Lynn, 2008; Enwere et al., 2014; Siegmund et al., 2023) is significantly induced by response to *cagA-*positive strains but not in *ΔcagA* mutants. Moreover, increased membrane-associated Fn14 expression in epithelial cells was closely associated with alterations in mucosal barrier integrity and epithelial remodeling (Chen et al., 2015; Acharya et al., 2019), suggesting a potential role for Fn14 as a hallmark of mucosal remodeling during gastric disease progression. Together, these findings suggest that CagA may exacerbate epithelial injury by promoting Fn14 expression and enhancing susceptibility to TNFR1-mediated apoptotic or necroptotic signaling. This Fn14 including mechanism may partly explain why *cagA*-positive *H. pylori* strains are associated with more severe gastric pathology, including atrophic gastritis and increased gastric cancer risk.

Although clinical biopsy samples are often heterogeneous and difficult to control, they remain important for confirming and complementing *in vitro* findings, as they can reveal tissue-level expression patterns, such as TWEAK, that are not detectable in simplified epithelial monoculture models such as AGS cells. In the present study, TWEAK expression was detected in whole gastric tissues but was hardly detected in infected AGS cells. In clinical biopsy samples, TWEAK expression correlated with IL-13 expression but not with Fn14 expression, *cagA* status, *H. pylori* infection status, or disease status. Interestingly, the relationship among TWEAK, Fn14, and IL-13 in clinical gastric tissues appeared highly complex rather than linear, suggesting context-dependent regulatory interactions. In previous studies conducted in other tissue contexts, such as glioma and dermal tissues, TWEAK has been shown to act as a potent inducer of Fn14 expression (Tran et al., 2005; Gu et al., 2023). This induction is mediated, at least in part, through activation of NF-κB signaling downstream of TWEAK–Fn14 ligand–receptor interaction, followed by direct binding of NF-κB to consensus elements within the Fn14 promoter region (Tran et al., 2006). However, in our *H. pylori* infection model using AGS cells, Fn14 expression was increased in a CagA-dependent manner despite minimal or undetectable TWEAK expression. This observation suggests that Fn14 upregulation in this context may occur independently of TWEAK signaling. One possible explanation is the involvement of the *cag* pathogenicity island (*cag*PAI), whereby translocated CagA activates NF-κB signaling following injection into host cells (Pachathundikandi et al., 2013), thereby promoting Fn14 expression. Further study is necessary to understand the alternative TWEAK-independent Fn14 induction pathways driven by *H. pylori* virulence factors.

**Figure 11:**
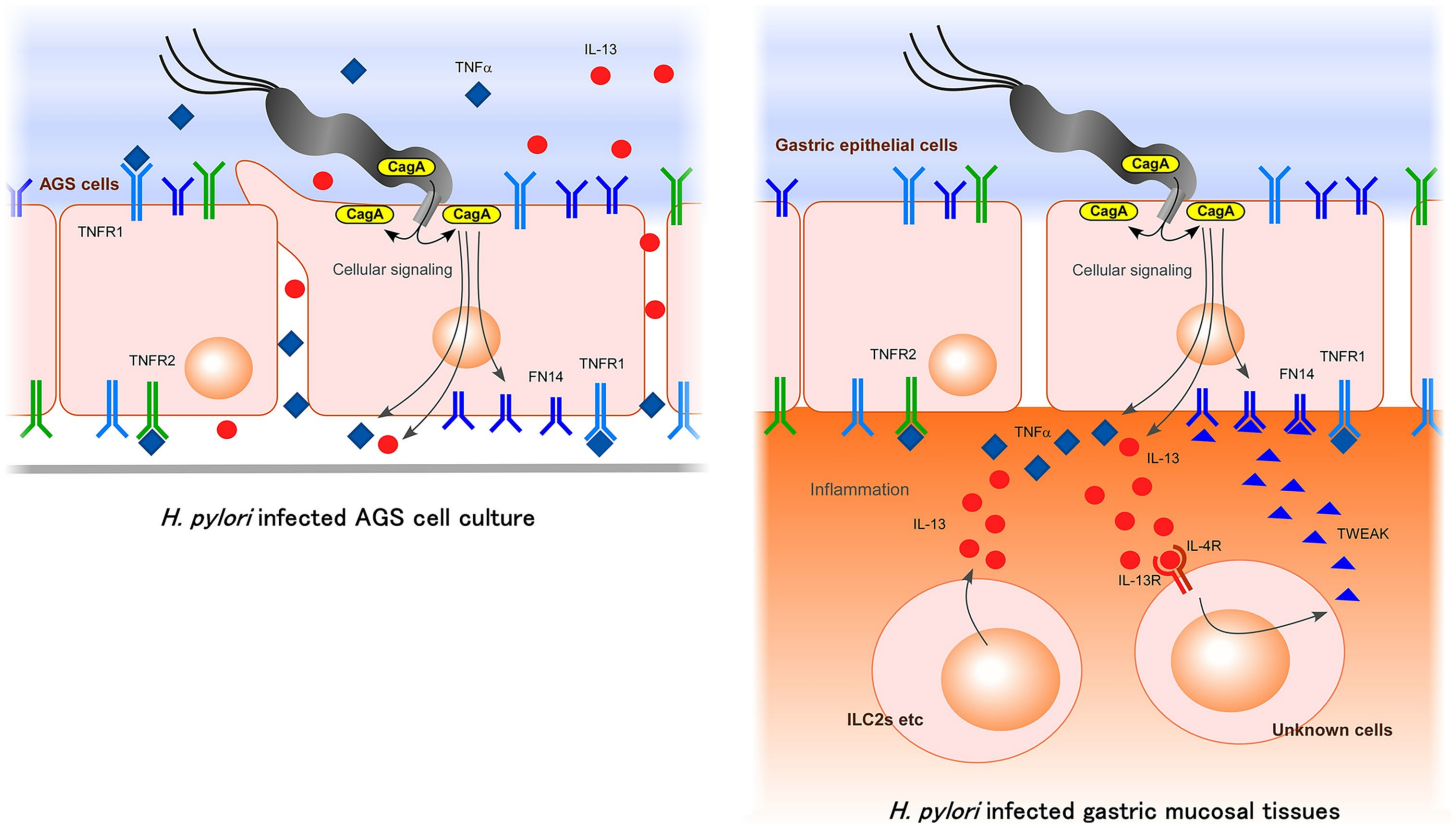
Schematic hypothetical representation of cytokine signaling and inflammatory pathway activation in *Helicobacter pylori*–infected gastric epithelial cells *in vitro* (left) and gastric mucosal tissue *in vivo* (right) from the gene expression analysis in this study.

Although this complexity complicates interpretation, it is biologically important and may provide further insight into gastric mucosal inflammation and remodeling. These findings also suggest that IL-13, produced by gastric epithelial cells and possibly also by immune cells within the gastric mucosa, may be associated with TWEAK expression in gastric tissue. However, a direct regulatory relationship cannot be established from the current data. IL-13 is a host-derived mediator of tissue response and immune regulation (Kawashima et al., 2006; Contreras-Panta et al., 2024). Such IL-13-competent immune populations are not represented in our *in vitro* epithelial monoculture, which may explain why the cagA-dependent induction of IL-13 observed in epithelial cells *in vitro* does not translate into a clear association with *H. pylori* or cagA status in clinical biopsies (Figure 11). In line with previous reports, IL-13 appears to be more closely linked to gastric mucosal remodeling and sequential histopathologic transitions, such as progression to atrophic gastritis and intestinal metaplasia, than to infection status alone (Marotti et al., 2008). This provides a biologically coherent explanation for the *in vitro*–*in vivo* differences observed in this study. Accordingly, IL-13 expression levels in whole-tissue samples likely reflect the intensity and nature of the inflammatory and reparative response, as well as structural alterations such as atrophy or metaplasia, rather than simply the presence of epithelial infection markers (Di Martino et al., 2019).

However, several limitations should be noted. First of all, our *in vitro* model relied on a gastric adenocarcinoma epithelial cell line and lacked key immune components—such as macrophages, T cells, and group 2 innate lymphoid cells (ILC2s)—that are recognized sources of cytokines, including IL-13 and TWEAK *in vivo*. Consequently, the cytokine regulation observed in this study likely reflects predominantly epithelial contributions to the host response. Furthermore, because AGS cells represent a single cell population, the specific cellular sources of individual cytokines could not be determined. Future studies employing more advanced approaches, such as single-cell transcriptomic analyses or *in vivo* spatial profiling, will be important to identify the distinct cell types responsible for producing cytokines such as TWEAK within the gastric mucosa. Second, the clinical cohort was derived from an Indonesian population with relatively low *H. pylori* prevalence and gastric cancer incidence, which may limit the generalizability of these findings to high-risk regions. The modest sample size (*n* = 85), including a limited number of intestinal metaplasia cases (*n* = 10), further reduces statistical power. Future studies should validate these findings in larger cohorts from high-incidence populations and incorporate immune–epithelial co-culture systems to define better the integrated signaling networks driving metaplastic transformation. Third, although transcriptomic analysis of mRNA expression is sensitive for detecting early cellular responses to biological stimuli such as *H. pylori* infection, it does not necessarily reflect protein abundance or functional activity. Protein levels are regulated by additional mechanisms, including translational efficiency, stability, secretion kinetics, and post-translational modifications. Therefore, the transcriptional changes observed in this study may not directly correspond to cytokine protein production or downstream signaling outcomes. Future investigations incorporating protein-level validation approaches will be important to clarify the functional significance of these transcriptional responses. Fourth, although the AGS infection model demonstrated CagA-dependent morphological and transcriptional changes, formal cytotoxicity assessments were not performed. Therefore, it remains unclear whether *H. pylori* infection induces epithelial cell damage under these conditions. The inclusion of cytotoxicity readouts would provide important functional evidence linking CagA activity to host-cell injury for future study. Lastly, the *cagA*-complemented strain derived from the lab-grown TN2 strain failed to show CagA phosphorylation after infection of AGS cells, despite the reinserted *cagA* gene being identical to that of the wild-type strain. It would be important to identify the deficient process in this strain that abrogates CagA phosphorylation and downstream CagA-dependent functional activity. This represents an important limitation of the present study and should be addressed in future investigations.

Our findings suggested that cagA-positive *H. pylori* infection is associated with increased Fn14 expression, whereas *H. pylori* infection in general is associated with reduced TNFR2 expression. While IL-13 signaling is more closely linked to mucosal injury and epithelial repair responses than to *H. pylori* infection or *cagA* presence alone, suggesting a dysregulated cytokine–receptor network. These findings indicate differential and context-dependent regulation of TNF receptor family members rather than a single unified cagA-driven mechanism.

## Data Availability

The raw data supporting the conclusions of this article will be made available by the authors, without undue reservation.
