# Supplementary material for: CagA-dependent expression of anti-inflammatory cytokine IL-13 and TNFRSF member Fn14 in Helicobacter pylori infected gastric cells and tissues
Source: Front Microbiol. 2026 Jun 16;17:1812604. doi: 10.3389/fmicb.2026.1812604 (PMC13314785; doi:10.3389/fmicb.2026.1812604)
Supplement: Supplementary file 1 [file Table_1.pdf]

Supplementary Material

1 Supplementary Tables

Supplementary Table S1| *Helicobacter pylori* strains used in this study

H. pylori strain	Description	Reference
TN2	Laboratory strain, original TN2GF4 was isolated from a Japanese gastric ulcer patient and used in Mongolian gerbil infection study, used in the experiments in Fig. 1 and 3.	Watanabe *et al* 1998
TN2Δ*cagA* (KmR)	3 single colony mixture of the strain that *cagA* ORF of TN2 was deleted and replaced with *aph* cassette (kmR), used in the experiments in Fig. 1 and 3.	This study
TN2 *cagA*-comp (CmR)	The *cagA* from strain TN2 was complemented into the same location in strain TN2Δ*cagA* genome with *cat* cassette (CmR) just downstream of *cagA*, used in the experiments in Fig. 1 and 3.	This study
TN2g2 S3S4	Two single colonies-mixture (S3S4) of second generation (g2) TN2 re-adapted and isolated from gerbil stomach (antrum), used in the experiments in Fig. 1 and 2.	This study Kubo *et al.* 2025
TN2g3 Δ*cagA* (KmR)	Gerbils stomach (antrum) adapted 4 single colonies mixture of Δ*cagA* strain that *cagA* ORF of strain TN2g2S3S4 was deleted and replaced with *aph* cassette (kmR), used in the experiments in Fig. 1 and 2.	This study Kubo *et al.* 2025
TN2g4 *cagA* comp (CmR)	Gerbil stomach (antrum) adapted 4 single colonies mixture of the *cagA*-complemented strain that *cagA* from strain TN2 was complemented into the same location in strain TN2g3Δ*cagA* genome with *cat* cassette (CmR) just downstream of *cagA,* used in the experiments in Fig. 1 and 2.	This study

Supplementary Table S2| Primers for constructing the *cagA* deletion (Δ*cagA*) mutant strain and the *cagA* complemented (*cagA*-comp) strain in genome of the Δ*cagA* strain according in Figure S1.

Primer name	DNA sequences	Target	Template DNA
Kmr-F1	gccacgttgtgtctcaaaatctctg	Km resistant cassette	pUC-4K plasmid (Amersham)
Km-R2	ttagaaaaactcatcgagcatcaaatga		
TN2cagA-F4	ggttgttatcactagccctaaagttactac	Upstream of *cagA*	TN2 genome
*KmrF1comp-*TN2cagA-R1	*cagagattttgagacacaacgtggc*ggtttctcctttaccatttggatttggtatcaaag		

KmrR2comp- TN2cagA-F3	*tcatttgatgctcgatgagtttttctaa*aggattaaggaatac caaaaacgcaaaaac	Downstream of *cagA*	TN2 genome
TN2cagA-R5	cattttcccttaatcgctcaccaaattgag		
TN2cagA-F5	gatgtcatcagagattttccaaatgtctgc	Upstream and downstream of *Δ cagA* and *cagA*-comp candidate	Genome of TN2 and transformants
TN2cagA-R6	atttgaatgtttcgcatttagccacttctc		
TN2cagA-F2	atgactaacgaaaccattgatcaaacaaca	*cagA* with start codon	TN2 genome
TN2cagA-R2	ttaagatttctggaaaccactttttgtagt	*cagA* with stop codon	TN2 genome
cat-F1	tctaaatgataacggaattccgtcgtcg	Cm resistant cassette	pBSC103 (Drs. Nakazawa, T, Berg, DE, Cover, TL)
cat-R2	ttatttattcagcaagtcttgtaattcatccaaaaaac		
catF1comp- TN2cagA-R2	*cgacgacggaattccgttatcatttaga*ttaagatttctgga aaccactttttgtagt	*cagA* upstream and *cagA* ORF	TN2 genome
catR2comp- TN2cagA-F3	*gttttttggatgaattacaagacttgctgaataaataa*agg attaaggaataccaaaaacgcaaaaac	Downstream of *cagA*	TN2 genome
TN2cagA-F8	caactttcttgtagctgtcagtgattcttgtaag	*cagA* upstream	TN2 genome and the *cagA*-comp fusion PCR product
TN2cagA-R7	gctattttatggagcattttgccctttcaacg	*cagA* downstream	

Supplementary Table S3| Primer sets for *H. pylori* qPCR

Primer name	Template DNA sequences*	Sequence	qPCR fragment size (bp)
ureB-sense23q<br>ureB-anti23q	TN2	ggtggtattgacacacacatccactt<br>gaatattcttcagccgctctgagcat	179
ureB-sense7q<br>ureB-anti8q	TN2	cgcttccactaaccccactatccctttc<br>cttttttgtttttgtcagctgtttgccaag	266
ureB-F1-Idn2<br>ureB-R1-Idn2	Indonesian consensus	gatcgctgaagtagaacatgactacacc<br>gtttttaacgccatcttgcatgtctttgtt	247
cagA-sense23q<br>cagA-anti23q	TN2	gctaacgcaaaaaaaccctgagtggc<br>tcaacttggtggaaaacttgaacgaatcagaataa	158
cagA-F1-Idn2<br>cagA-R1-Idn2	Indonesian consensus	atttcagcaaggcagaagaaacgctaaaag<br>aggttttcaatttttgaaatccattctgga	211

Supplementary Figure

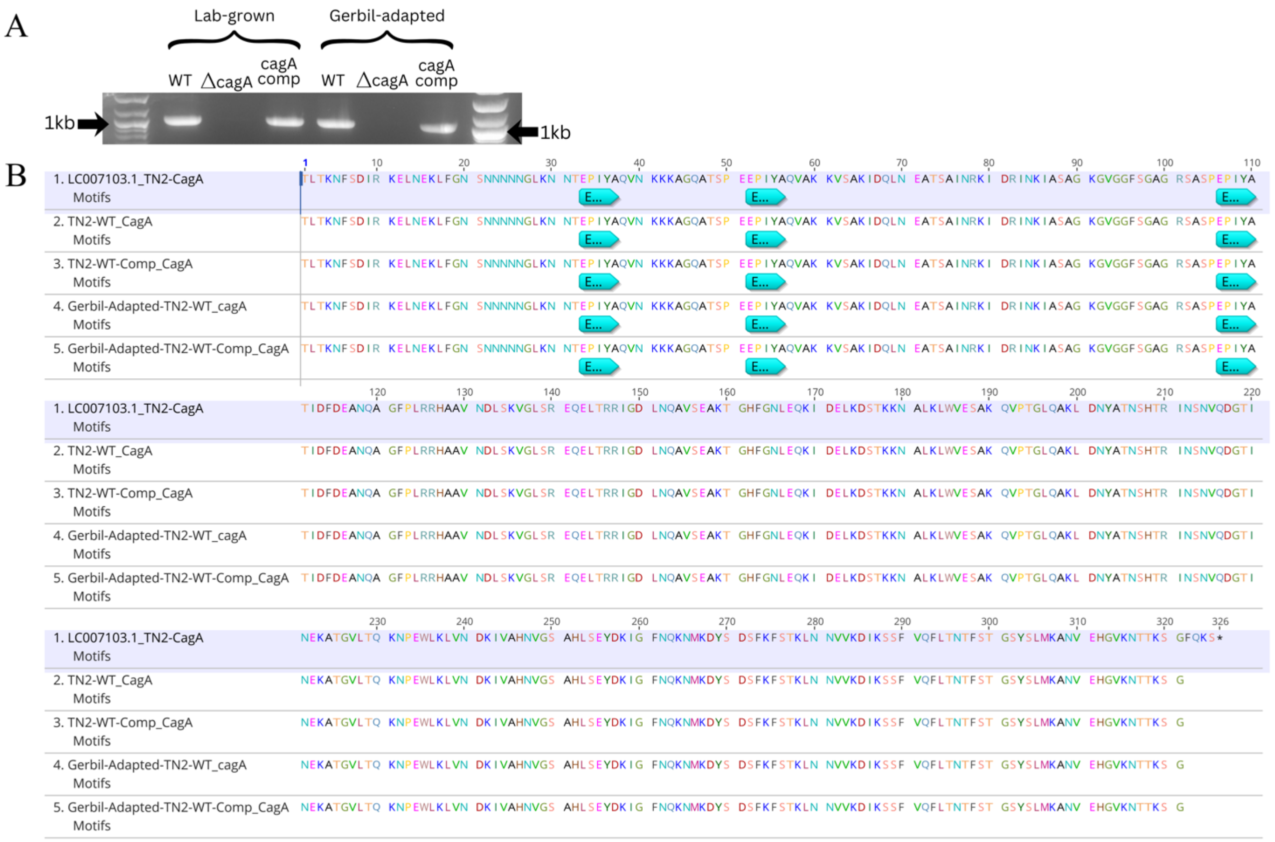

Supplementary Figure S1| Confirmation of *cagA* deletion and complementation and sequence conservation across strains. (**A**) PCR analysis confirming the presence or absence of *cagA* in wild-type (WT), Δ*cagA*, and *cagA*-complemented (comp) strains from both laboratory-grown and gerbil-adapted TN2 backgrounds. The *cagA* amplicon is detected in WT and complemented strains, but absent in Δ*cagA* mutants, confirming successful deletion and re-insertion. (**B**) Amino acid sequence alignment of CagA from laboratory-grown and gerbil-adapted WT and complemented strains, together with the reference genome (LC007103.1). Conserved EPIYA motifs are highlighted in cyan arrow. The alignment shows complete conservation of the *cagA* sequence among all *cagA*-positive strains, confirming that phenotypic differences are not attributable to sequence variation within *cagA*.
